# Molecular-driven lead-free halide photoferroelectric solid solution for high-temperature sensitive self-powered x-ray detection

**DOI:** 10.1126/sciadv.aef7693

**Published:** 2026-08-21

**Authors:** Lijun Xu, Yao Li, Qianwen Guan, Hang Li, Chengshu Zhang, Huang Ye, Haiqing Zhong, Chengmin Ji, Zhenyue Wu, Lina Li, Junhua Luo

**Affiliations:** ^1^ State Key Laboratory of Functional Crystals and Devices, Fujian Institute of Research on the Structure of Matter, Chinese Academy of Sciences, Fuzhou, Fujian 350002, P.R. China.; ^2^ Fujian Science and Technology Innovation Laboratory for Optoelectronic Information of China, Fuzhou, Fujian 350108, P.R. China.; ^3^ University of Chinese Academy of Sciences, Chinese Academy of Sciences, Beijing 100049, P.R. China.

## Abstract

Halide double perovskite ferroelectrics have recently emerged as an environmentally friendly candidate in radiation detection, photovoltaics, and optoelectronic memory devices due to their unique spontaneous polarization and semiconductor properties. However, it is a huge challenge to achieve efficient carrier transport and high Curie temperature (*T*_c_) in double perovskite ferroelectrics, owing to the limitations of twisted frameworks and structural construction. Here, we present a high-*T*_c_ heterovalent metal solid solution double perovskite ferroelectric, (4Br2FBZ)_2_CsAgBiBr_7_ (4Br2FBZ-CAB, 4Br2FBZ = 4-bromo-2-fluorobenzylammonium), through a strategy of cation-engineered symmetry regulation. The metal ions Ag and Bi transitioned from an alternating arrangement to a mixed-site solid solution pattern, effectively optimizing the electronic band structure to enhance carrier transport. Meanwhile, 4Br2FBZ-CAB exhibits a high *T*_c_ of 484 kelvin, substantially expanding the family of high-*T*_c_ lead-free ferroelectrics. Benefiting from its remarkable photoelectric and ferroelectric properties, a high self-powered x-ray detection sensitivity of 273.8 microcoulomb per gray per square centimeter (μC Gy_air_^−1^ cm^−2^ ) was achieved at room temperature. In addition, this exceptionally high *T*_c_ enables the detector to operate self-powered at high temperatures, resulting in an excellent sensitivity of 896.9 μC Gy_air_^−1^ cm^−2^ and a low detection limit of 13.9 nGy_air_ s^−1^ at 425 kelvin. This work opens an avenue for expanding the family of high-*T*_c_ lead-free perovskite ferroelectrics with superior semiconducting properties.

## INTRODUCTION

Organic-inorganic hybrid perovskites (OIHPs) have garnered substantial interest due to their unique physical properties and high structural design flexibility, highlighting their great potential in applications such as ferroelectricity, photovoltaics, lasing, radiation detection, etc. (*1*–*6*). In particular, emerging OIHP photoferroelectric semiconductors hold importance in sensing, photodetection, data storage, and various life and military applications (*7*–*12*). Despite substantial progress in two-dimensional (2D) lead-based perovskite ferroelectrics, the practical application of these materials necessitates addressing the critical issue of toxic lead. Notably, halogen double perovskites have emerged as a promising class of lead-free semiconductors with diverse applications, including photovoltaics, photocatalysis, radiation detection, and light emitting (*13*–*17*). Since the 1930s (*18*), halogen double perovskites have seen continuous development and remarkable achievements, such as Cs_2_AgBiBr_6_ (*19*), Cs_2_AgInCl_6_ (*20*), and Cs_2_NaBiCl_6_ (*21*). However, the highly symmetric 3D structure prevents the emergence of ferroelectricity, which has driven the development of 2D double perovskite ferroelectrics (*15*, *22*, *23*). Reducing the dimensionality to 2D by introducing directionally aligned organic cations to induce symmetry breaking, thereby achieving ferroelectricity, emerges as an effective strategy. The general formula for 2D bilayered double perovskites is A_2_BM^I^M^III^X_7_, where A is an organic cation, B is a small cation located within the octahedral cavities, M^I^ and M^III^ are heterovalent metals, and X is a halogen (*24*–*27*). For example, 2D double perovskite ferroelectric, (benzylammonium)_2_CsAgBiBr_7_, exhibits a high Curie temperature (*T*_c_) of 483 K (*28*). However, the large lattice distortion induced by the radius difference of heterovalent metal ions and the enhanced carrier scattering caused by greater electronic potential fluctuations due to disparities in electronic structure both result in poor carrier mobility and severely degrade the photoelectric conversion efficiency (*25*, *29*–*32*). This severely limits the application of 2D double perovskites in photodetection. Therefore, it is imperative to develop 2D double perovskites with high *T*_c_ and excellent semiconductor properties.

In general, the complex elemental composition and specific synthesis conditions of double perovskites lead to deep-level defects such as antisite defects and vacancies, which constrain the efficient transport of charge carriers (*33*–*35*). Precise control of the metal element stoichiometric ratio in precursors to synthesize high-quality single crystals, along with defect passivation through additives, is effective means to reduce defects (*36*, *37*). In addition, metal ions are decisive factors in the electronic band structure of perovskites. Metal doping serves as an effective strategy to modulate electronic structure and enhance charge transport. Recently, Jia *et al.* (*38*) revealed that the solid solution of two metal ions adjusts the electronic structure and reduces structural distortion, thereby enhancing carrier transport efficiency and achieving a photoconversion ratio greater than 100. However, their failure to successfully induce ferroelectricity greatly limits the material’s application in the field of optoelectronics. In general, ion co-occupancy is influenced by factors such as structural symmetry, lattice strain energy, and organic cations (*39*). On the basis of “ferroelectrochemical” theory, the modification of organic cations has proven highly effective in inducing ferroelectric ordering through symmetry breaking and regulating the *T*_c_ (*40*). This provides insight into the solid solution of metal ions in double perovskite induced by regulating lattice symmetry. Inspired by this, effective cation design can not only efficiently construct ferroelectrics with high *T*_c_ but also induce solid solution of metal ion in double perovskites, thereby improving their semiconductor properties and enabling high-performance radiation detectors.

**Fig. 1.**
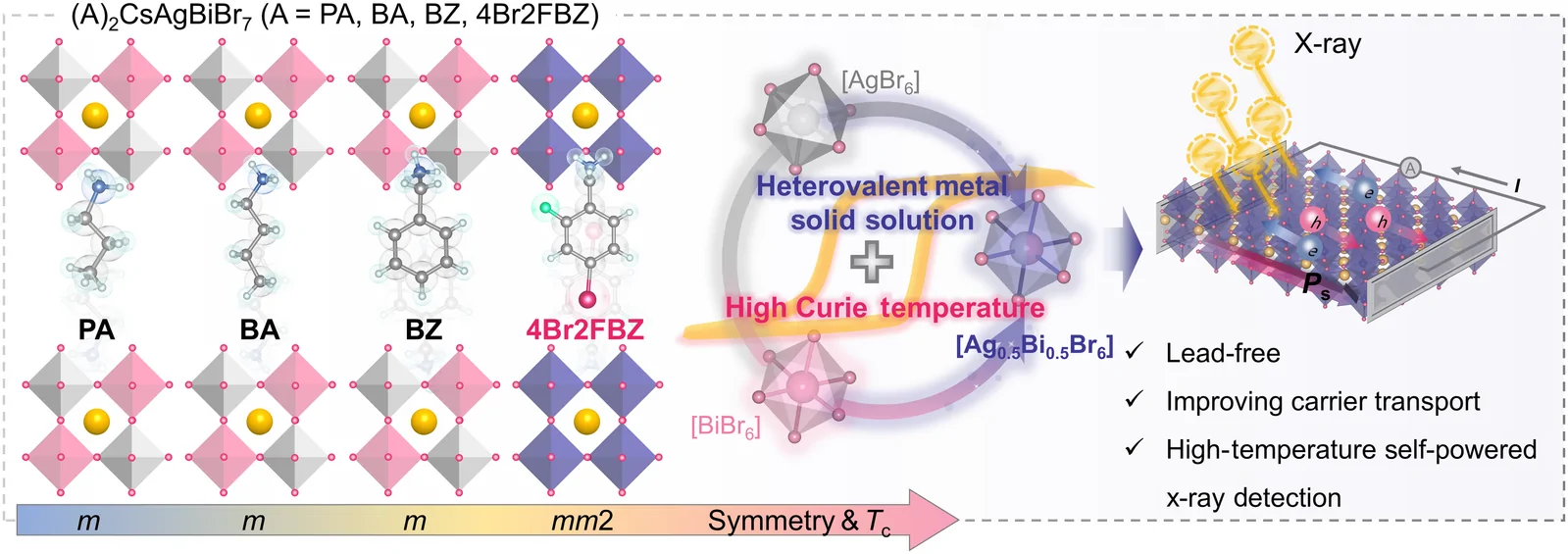
Material design strategy. Design of heterovalent metal solid solution multilayered double perovskite ferroelectric with high *T*_c_ to achieve high-performance photodetection with a large temperature window.

In this work, by cation engineering (Fig. 1), we designed a 2D double perovskite ferroelectric, (4Br2FBZ)_2_CsAgBiBr_7_ (4Br2FBZ-CAB, 4Br2FBZ = 4-bromo-2-fluorobenzylammonium), which exhibits a high *T*_c_ and solid solution of heterovalent metal ion. The tailoring of organic cations drives the transition of structural symmetry inducing the formation of a heterovalent metal solid solution. The metal ions Ag and Bi changed from an alternating arrangement to a mixed-site solid solution pattern, effectively optimizing the electronic band structure to enhance carrier transport. Meanwhile, the aligned aromatic cation also induces ferroelectricity in 4Br2FBZ-CAB, which exhibits a high *T*_c_ of 484 K, substantially expanding the family of high-*T*_c_ lead-free ferroelectrics. Benefiting from its remarkable photoelectric and ferroelectric properties, a high self-powered x-ray detection sensitivity of 273.8 μC Gy_air_^−1^ cm^−2^ was achieved at room temperature. In addition, this exceptionally high *T*_c_ enables the detector to operate self-powered at high temperatures, resulting in an excellent sensitivity of 896.9 μC Gy_air_^−1^ cm^−2^ and a low detection limit of 13.9 nGy_air_ s^−1^ at 425 K. This work opens an avenue for expanding the family of high-*T*_c_ lead-free perovskite ferroelectrics with superior semiconducting properties, enabling efficient self-powered x-ray detection.

## RESULTS

### Structural analyses

**Fig. 2.**
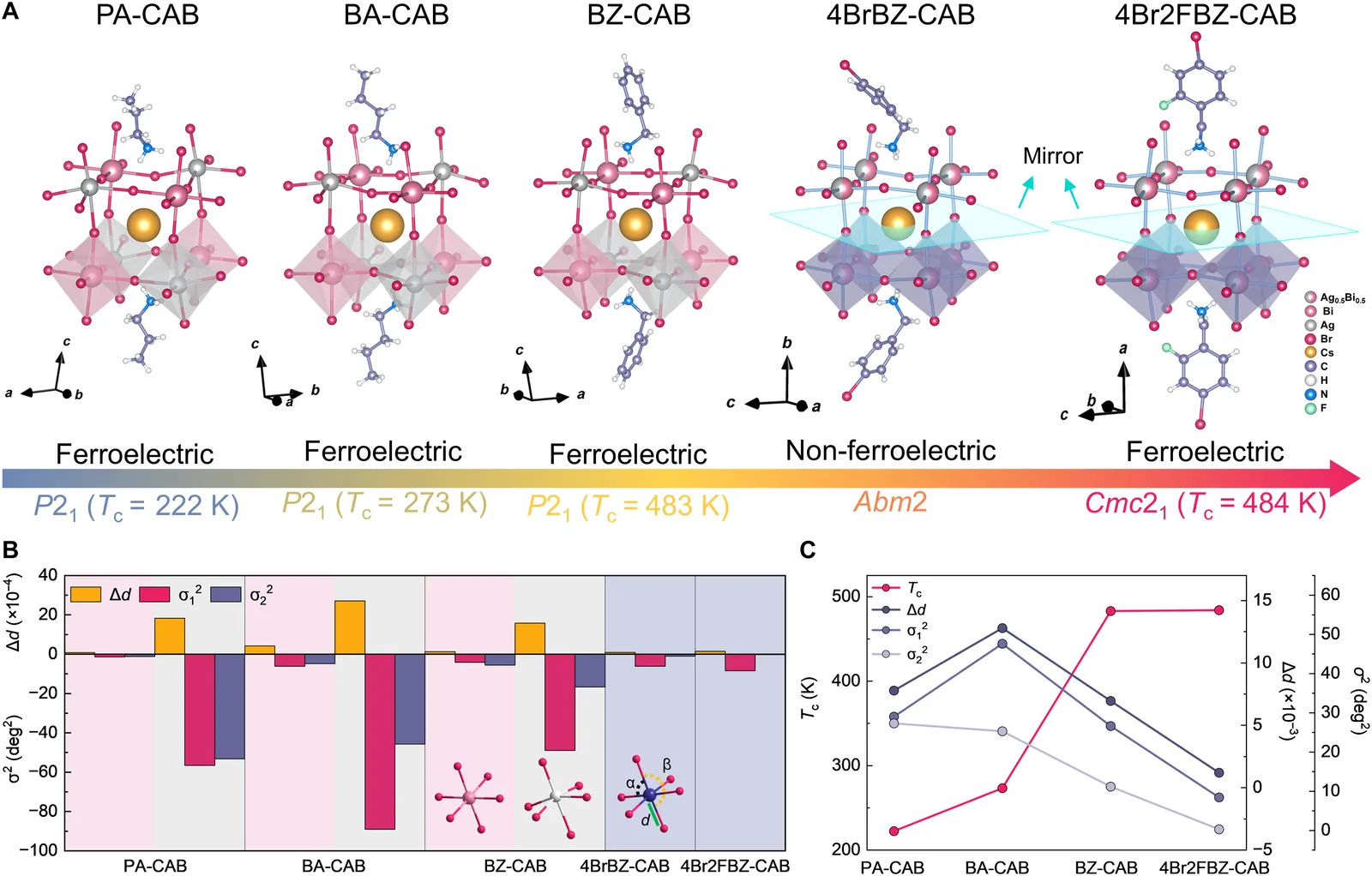
Crystal structure analyses of the series of double perovskite compounds. (**A**) Structures of (n-propylammonium)_2_CsAgBiBr_7_ (PA-CAB), (n-butylammonium)_2_CsAgBiBr_7_ (BA-CAB), (benzylammonium)_2_CsAgBiBr_7_ (BZ-CAB), (4-bromobenzylammonium)_2_CsAgBiBr_7_ (4BrBZ-CAB), and (4-bromo-2-fluorobenzylammonium)_2_CsAgBiBr_7_ (4Br2FBZ-CAB). (**B**) Calculated octahedral bond angle variance parameters (σ_1_^2^ and σ_2_^2^) and bond length distortion parameter (Δ*d*). (**C**) Changes in *T*_c_, bond angle, and bond length variance for the series of compounds.

2D lead-free double perovskite motif of (A)_2_CsAgBiBr_7_, where A = PA (n-propylammonium), BA (n-butylammonium), BZ (benzylammonium), 4BrBZ (4-bromobenzylammonium), 4Br2FBZ (4-bromo-2-fluorobenzylammonium), is constructed by alloying organic spacers into the 3D prototypical perovskite Cs_2_AgBiBr_6_ (*26*, *28*, *41*, *42*). Structurally, organic cations exhibit adjustability, which play an important role in regulating the structural motifs and properties of 2D hybrid double perovskites. To deeply understand the effect of organic cations on 2D hybrid double perovskite structures, we synthesized a series of double perovskite single crystals from aqueous solutions with stoichiometric ratios. Their phase purity was confirmed by powder x-ray diffraction (PXRD) in fig. S1. As shown in Fig. 2A, single-crystal structure analysis demonstrate that those compounds crystallize in the polar point group of 2 or *mm*2 at low temperature phase (tables S1 to S3). The basic structure is composed of alternately arranged bilayered inorganic sheets and organic spacers, which are linked to each other by N-H···Br hydrogen bonds (fig. S2). Furthermore, the consistent orientation of organic cations along specific axes, combined with the twisting of the inorganic components, drives the formation of long-range ferroelectric order. It is observed that the compounds constructed by aliphatic cations, such as PA-CAB and BA-CAB, exhibit Curie temperature (*T*_c_) below room temperature, which severely limits the further application of these materials. Notably, replacing aliphatic cations with aromatic cations, such as BZ, can effectively increase the phase transition barrier and raise the *T*_c_.

Unfortunately, the Ag and Bi ions in compounds PA-CAB, BA-CAB, and BZ-CAB occupy distinct sites, leading to strong structural distortion that impedes efficient carrier transport. Furthermore, regulating structural symmetry through cation design holds promise for achieving a mixed-site mode between Ag and Bi ions. The para-bromination of BZ via the heavy atom effect synthesized compound 4BrBZ-CAB. The compound exhibits enhanced symmetry, crystallizing in the *mm*2 point group, with Ag and Bi ions adopting a co-occupancy mode (the occupancy rates of Ag^+^ and Bi^3+^ is 0.5:0.5). This model of solid solution for heterovalent metals effectively reduces structural distortion and optimizes the electronic band structure. However, the highly tilted, parallel alignment of the cations prevented dynamic rotation, thus failed to induce ferroelectricity. Therefore, by further regulating the arrangement of cations, compound 4Br2FBZ-CAB was synthesized using the 4Br2FBZ cation with ortho-fluorine substitution and para-bromine substitution. Because of interactions between the F substituent and the inorganic framework as well as adjacent cations (such as F···Br and F···π interactions), the 4Br2FBZ cation adopts an arrangement similar to that of the BZ cation (fig. S3). Unexpectedly, compound 4Br2FBZ-CAB also crystallized in the *mm*2 polar point group, exhibiting ferroelectricity with a high *T*_c_ while adopting a mixed-site mode for Ag and Bi ions. Refinement was performed using different metal ion occupancy models, and the results showed that the 0.5:0.5 mixed occupancy model provided a statistically preferred description (table S4). Structurally, we further analyzed the causes for this change. The parameters octahedral bond length distortion (Δ*d*) and bond angle variance (σ_1_^2^ and σ_2_^2^) describe octahedral distortion: $∆d=\frac{1}{6}\sum_{i=1}^{6}{\left(\frac{d_{i}-d}{d}\right)}^{2}$, $\sigma_{1}^{2}=\frac{1}{11}\sum_{i=1}^{12}{\left(\alpha_{i}-90\right)}^{2}$ and $\sigma_{2}^{2}=\frac{1}{2}\sum_{i=1}^{3}{\left(\beta_{i}-180\right)}^{2}$, where *d*_i_ and *d* denote the individual and mean Ag-Br or Bi-Br bond lengths, whereas α_i_ and β_i_ represent the Br-Ag-Br or Br-Bi-Br angles between neighboring and non-neighboring Ag-Br or Bi-Br bonds, respectively (as illustrated in Fig. 2B, inset) (*43*). As shown in Fig. 2B, compounds adopting the split-site models exhibit large structural distortion, which is particularly exhibited in the [AgBr_6_] octahedron. For example, in the compound PA-CAB, the Δ*d* values for the [AgBr_6_] and [BiBr_6_] octahedra are 1.83 × 10^−3^ and 7.71 × 10^−5^, respectively, while the values for σ_1_^2^ and σ_2_^2^ are 56.58 and 53.29 and 1.39 and 1.23, respectively. In contrast, the model of solid solution for heterovalent metals avoids structural distortion caused by differences in ionic radii, resulting in a flatter inorganic framework. In compound 4Br2FBZ-CAB, the Δ*d*, σ_1_^2^, and σ_2_^2^ values for the [Ag_0.5_Bi_0.5_Br_6_] octahedron is 1.46 × 10^−4^, 8.39, and 0.22, respectively.

Organic cations play a crucial role in regulating structural symmetry, ferroelectricity, and Curie temperature. As shown in fig. S4, the organic spacer layer spacing expands from 6.80 Å in PA-CAB to 12.29 Å in 4Br2FBZ-CAB as the cation volume increases. Further analysis of the interactions between organic cations reveals that the organic layers in PA-CAB, BA-CAB, and BZ-CAB are connected solely through van der Waals interactions (fig. S5). In contrast, the organic layers in 4BrBZ-CAB are connected via Br···π interactions arising between the organic cations. It is worth noting that the organic layers in 4Br2FBZ-CAB feature both Br···π interactions and Br···Br halogen bonds (fig. S6). Furthermore, the F atoms of the 4Br2FBZ cations participate in F···π interactions with neighboring cations, as well as F···Br halogen interactions with the inorganic framework. Therefore, the halogen atoms on the cations introduce abundant interactions that constrain the swinging of the inorganic framework and reduce distortion, representing a crucial factor in the improvement of lattice symmetry. Furthermore, we analyzed the relationship between the structure’s symmetry and the metal ion occupation patterns. It was found that the compounds based on PA, BA, and BZ organic cations all crystallize in the monoclinic system with the point group 2. This low symmetry tends to cause the two metal ions to occupy distinct split sites. However, both compounds 4BrBZ-CAB and 4Br2FBZ-CAB crystallize in the orthorhombic crystal system with point group *mm*2, exhibiting crystallographic *m*-mirrors parallel and crossing the inorganic framework (fig. S7). In comparison, the lattice symmetry is improved, allowing Ag and Bi ions to occupy mixed sites. Furthermore, when the high-temperature phases (HTPs) of compounds PA-CAB, BA-CAB, and BZ-CAB crystallize in the low-symmetry monoclinic system with the space group *P*2_1_/*m*, Ag and Bi ions still adopt a split-site occupation. However, when the HTP crystallizes in the high-symmetry tetragonal system with the space group *I*4/*mmm*, these two metal ions adopt a mixed-site pattern (fig. S8). Similar rules are also observed in single-layer double perovskites, summarized in table S5. This strongly demonstrates that the lattice symmetry can be effectively altered by designing cations to regulate the occupancy pattern of metal ions. As shown in Fig. 2C, compound 4Br2FBZ-CAB exhibits a high *T*_c_, while the solid solution of Ag and Bi ions greatly reduces structural distortion, thereby facilitating carrier transport and substantially advancing the development of 2D lead-free ferroelectrics.

### Elemental analyses

**Fig. 3.**
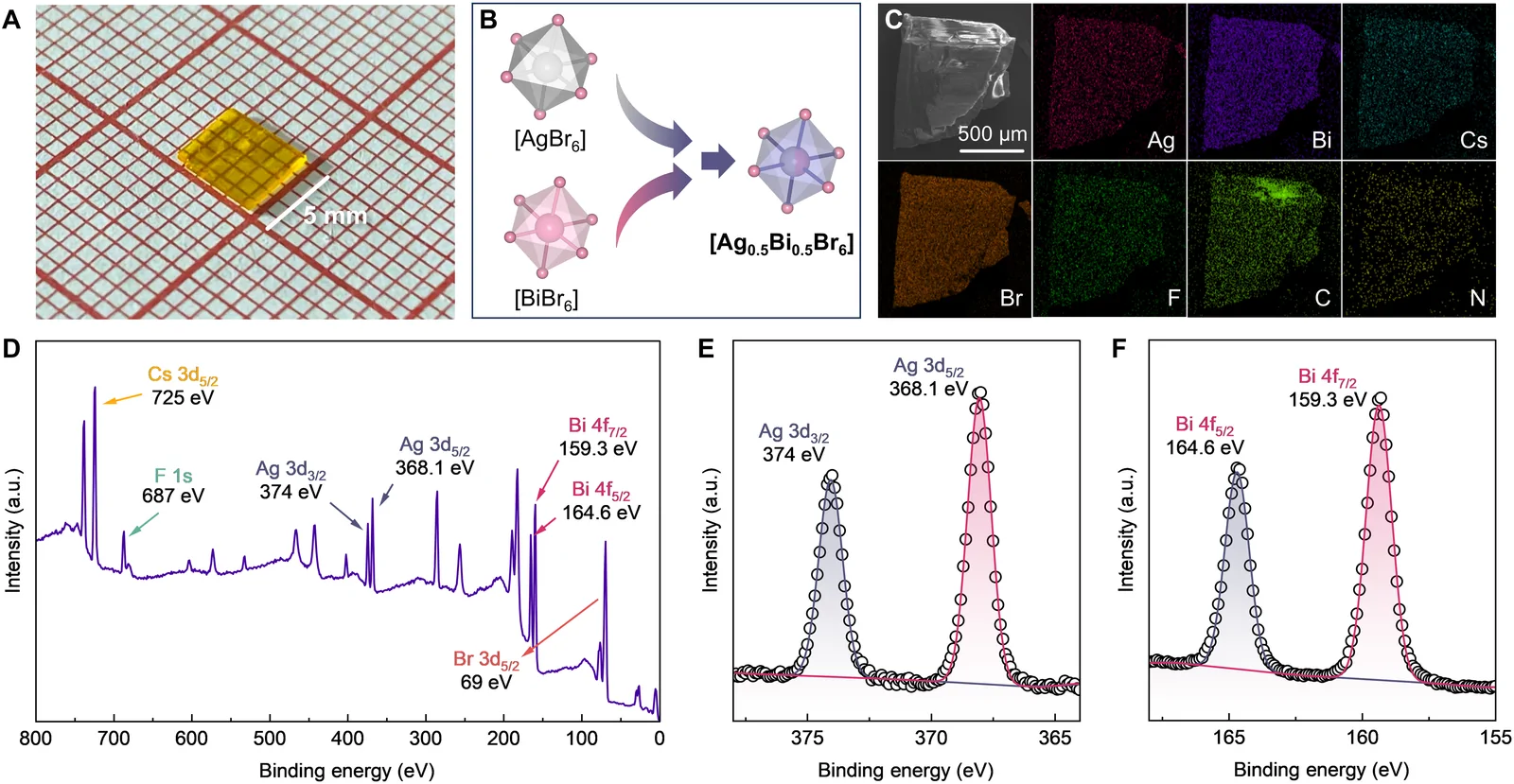
Crystal preparation and elemental analyses. (**A**) The photograph of a high-quality SC of 4Br2FBZ-CAB. (**B**) Equal ratio solid solution of Ag and Bi metal ions. (**C**) EDS element mapping images for 4Br2FBZ-CAB. XPS spectra of 4Br2FBZ-CAB (**D**), Ag 3d (**E**), and Bi 4f (**F**). a.u., arbitrary units.

Yellow block crystals of 4Br2FBZ-CAB were obtained by slowly cooling its solution in hydrobromic acid (Fig. 3A). Ag and Bi metal ions form a solid solution in equal proportions (Fig. 3B). As shown in Fig. 3C, the energy dispersive x-ray spectroscopy (EDS) scan results revealed that Ag and Bi elements are uniformly distributed in the structure, confirming the solid solution of metal ions. Furthermore, the x-ray photoelectron spectroscopy (XPS) results in Fig. 3 (D to F) indicate the presence of Ag and Bi elements within the crystal. The valence states of Ag and Bi in this structure are Ag^+^ (Ag 3d_5/2_ ∼ 368.1 eV and Ag 3d_3/2_ ∼ 374 eV) and Bi^3+^ (Bi 4f_7/2_ ∼ 159.3 eV and Bi 4f_5/2_ ∼ 164.6 eV), respectively, consistent with the expected chemical states. This systematically characterizes the solid solution properties of the heterovalent metals Ag and Bi in 4Br2FBZ-CAB.

### Phase transition and ferroelectric properties

**Fig. 4.**
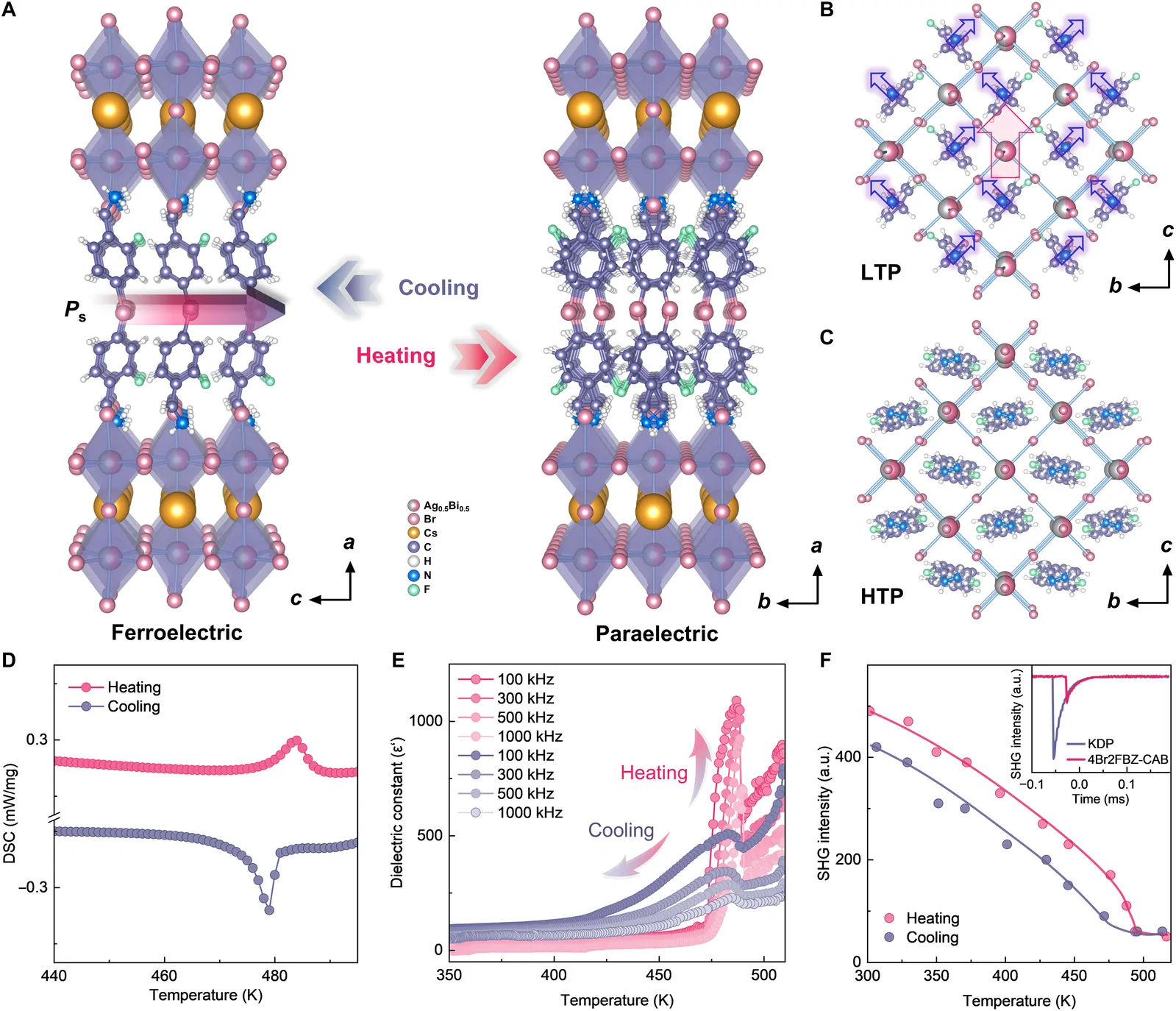
Crystal structure packing diagram and phase transition behavior. (**A**) The packing structures at LTP and HTP. (**B** and **C**) Interlayer cation arrangement in LTP (B) and HTP (C). (**D**) DSC curves of 4Br2FBZ-CAB during heating and cooling cycle. (**E**) The temperature-dependent dielectric constants. (**F**) The variable-temperature SHG intensities. Inset: The comparison of SHG signals of 4Br2FBZ-CAB and KDP.

**Fig. 5.**
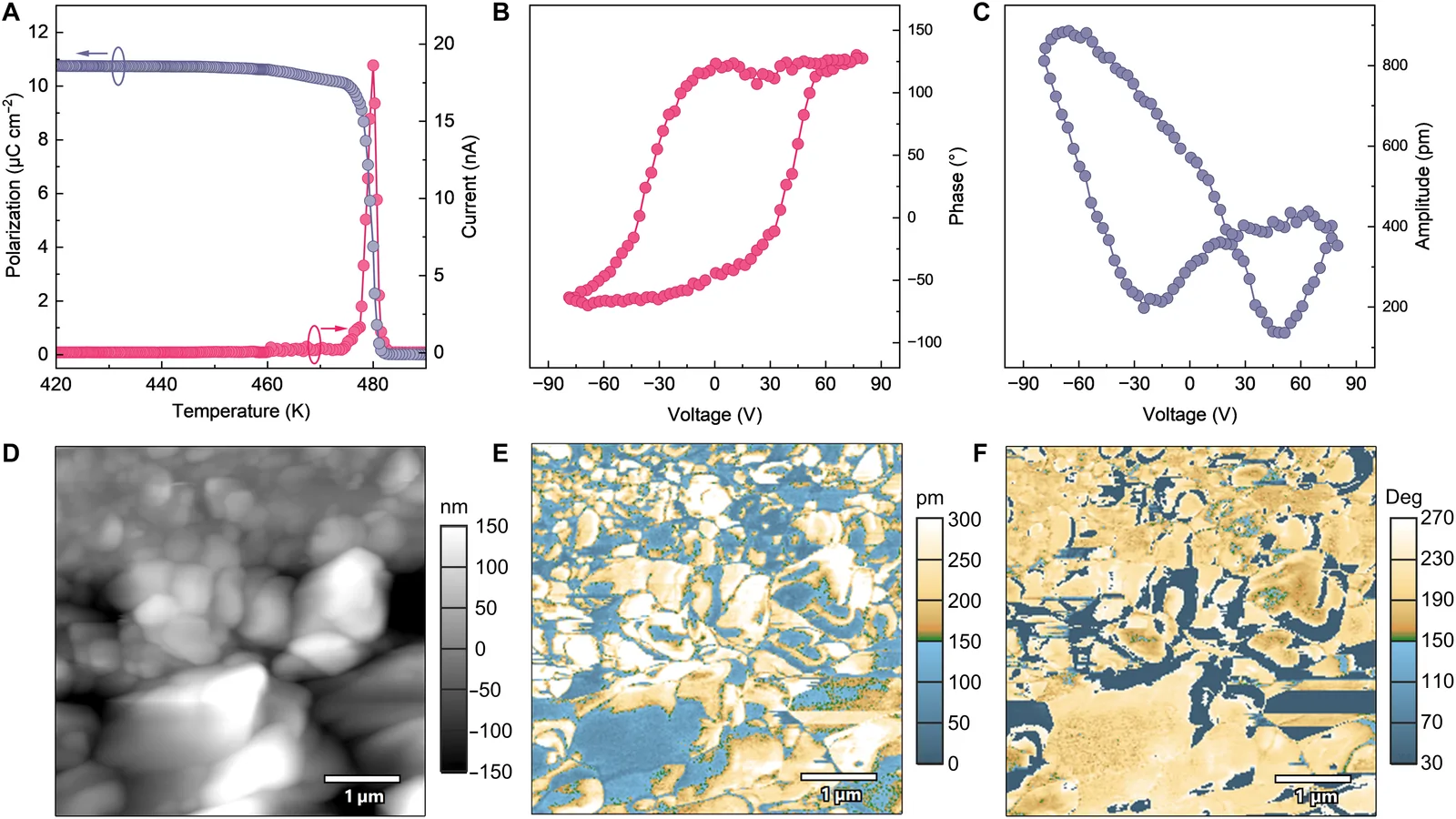
Ferroelectric property characteristic. (**A**) Temperature dependence of spontaneous polarization integrated from pyroelectric current. (**B** and **C**) The local PFM phase hysteresis (B) and amplitude butterfly loops (C). (**D**) The topography image. The corresponding PFM amplitude (**E**) and phase (**F**) image.

As depicted in Fig. 4A, compound 4Br2FBZ-CAB features a double-layer structure of organic cations and inorganic frameworks stacking alternately along the crystallographic *a* axis, with the polarization oriented along the *c* axis in low-temperature phase (LTP). Thermogravimetric analysis indicates that it is thermally stable up to ∼527 K (fig. S9). Subsequently, we attempted to record the HTP structure at 500 K. However, because of the extremely high temperature approaching the decomposition point of the compound, the crystal structure data were not ideal. Nevertheless, the inorganic framework shows high symmetry, and the organic cations are disordered, which cancels the polarization. In the LTP, organic cations adopt a T-shaped arrangement, with organic amines uniformly oriented to induce polarization (Fig. 4B). In the HTP, organic cations no longer maintain this ordered T-shaped arrangement, leading to the disappearance of polarization (Fig. 4C). A pair of exothermic/endothermic peaks were found by differential scanning calorimetry (DSC) measurements, suggesting that 4Br2FBZ-CAB goes through a reversible phase transition around 484 K (Fig. 4D). Moreover, the marked reversible dielectric anomalies at different frequencies are clearly observed, which show the sharp peak-like dielectric anomalies around *T*_c_ (Fig. 4E). These dielectric behaviors indicate the emergence of the potential electric ordering of 4Br2FBZ-CAB. Furthermore, the variable temperature behavior of second harmonic generation (SHG) signals was investigated for 4Br2FBZ-CAB (Fig. 4F). At room temperature, the SHG signal of 4Br2FBZ-CAB is about 0.3 times that of KH_2_PO_4_ (KDP) (Fig. 4F, inset). As the temperature increases, the SHG intensity gradually decreases, and the SHG signal becomes nearly undetectable near the *T*_c_, indicating a structural transition from noncentrosymmetric to centrosymmetric. The pyroelectric current was recorded during the cooling process, revealing a sharp pyroelectric current peak near the *T*_c_ (Fig. 5A). This further indicates the occurrence of a phase transition process, with a calculated spontaneous polarization (*P*_s_) of ∼10.75 μC cm^−2^. Piezoresponse force microscopy (PFM) measurement was conducted on 4Br2FBZ-CAB over an area of 5 μm by 5 μm to demonstrate its ferroelectric properties (Fig. 5D). As shown in Fig. 5 (E and F), the phase and amplitude images clearly reveal the ferroelectric domains in the 4Br2FBZ-CAB sample with a 180° phase contrast. The phase curve also indicates a phase difference of 180° (fig. S10). Subsequently, we selected a single domain region to investigate the polarization switching behavior of the ferroelectric domain. The phase-voltage curve exhibits distinct hysteresis, while the amplitude-voltage curve displays a characteristic butterfly shape, indicating the reversal of ferroelectric polarization (Fig. 5, B and C). To further visualize the domain switching process, we performed local domain manipulation experiments on 4Br2FBZ-CAB single-crystal thin films (fig. S11). As shown in fig. S10C, the pristine state of 4Br2FBZ-CAB exhibits a fragmented domain structure with distinct phase contrast. When a tip under 100-V bias was applied to pole the sample surface, yellow domains predominate, indicating that the polarization direction in some regions has been switched upward (fig. S11f). Meanwhile, the surface morphology of the sample remains unchanged (fig. S11, A and D). All these PFM results provide conclusive evidence for stable and switchable polarization in 4Br2FBZ-CAB.

### Optical and semiconductor properties

**Fig. 6.**
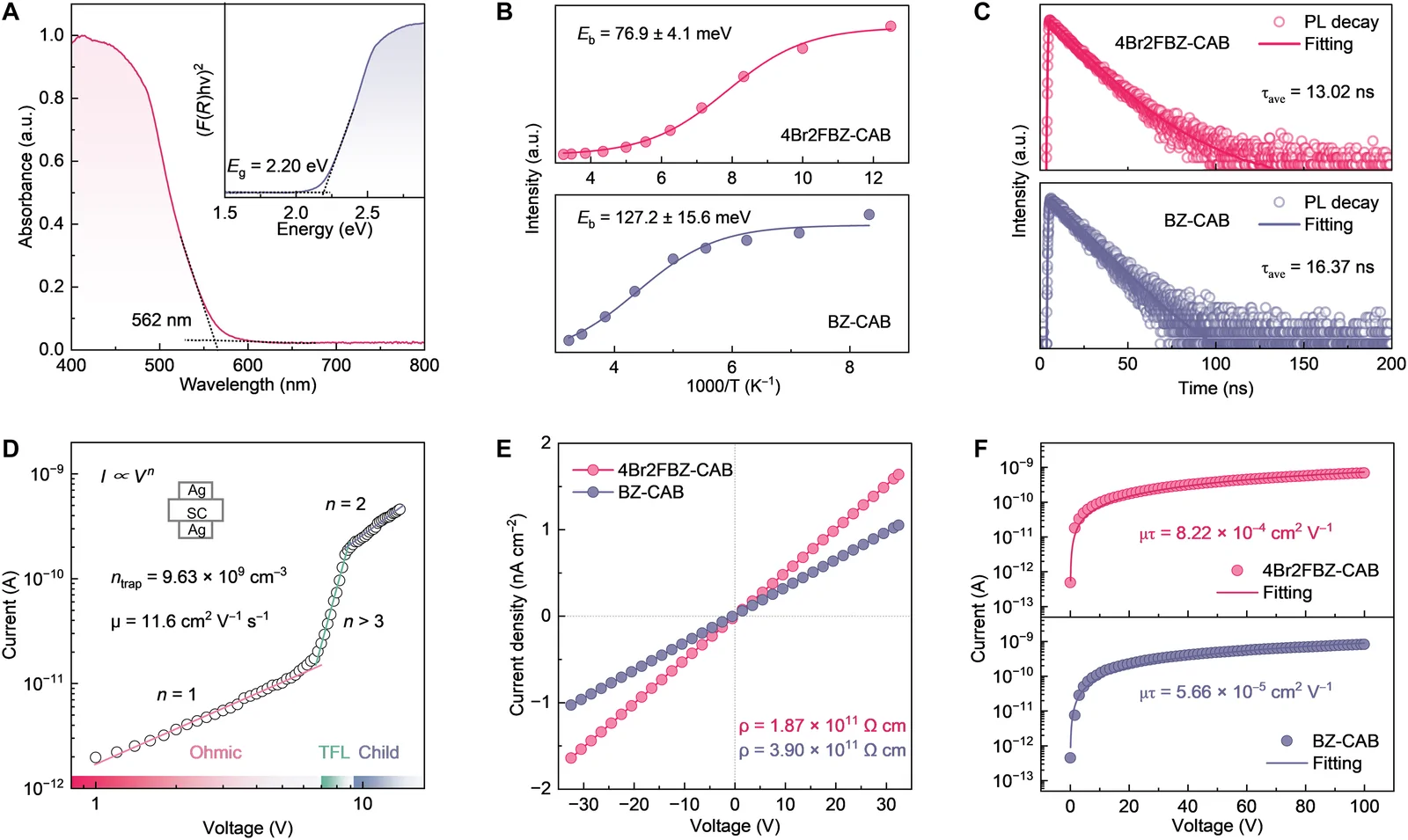
Optical properties and semiconductor characteristics. (**A**) Optical absorption spectrum with the corresponding Tauc plot (inset). (**B**) The exciton binding energies of the 4Br2FBZ-CAB and BZ-CAB. (**C**) PL decay curves of 4Br2FBZ-CAB and BZ-CAB under 450-nm excitation. (**D**) The SCLC analysis of the 4Br2FBZ-CAB SC device. (**E**) The resistivity of 4Br2FBZ-CAB and BZ-CAB SC. (**F**) Bias-dependent photoconductivity curves.

Furthermore, we evaluated the optical properties and semiconductor characteristics of 4Br2FBZ-CAB. The ultraviolet-visible (UV-vis) absorption spectrum and corresponding Tauc plot in Fig. 6A indicate that 4Br2FBZ-CAB has an absorption edge at 562 nm with a bandgap (*E*_g_) of 2.20 eV. Moreover, the band structure in fig. S12 was calculated using the density functional theory, showing that 4Br2FBZ-CAB exhibits a direct bandgap feature, and the calculated *E*_g_ of 2.37 eV coincides with the experimental result. In general, a low exciton binding energy (*E*_b_) promotes the rapid separation of electrons and holes for efficient extraction, leading to high-efficiency photoelectric conversion. As shown in Fig. 6B, temperature-dependent photoluminescence (PL) spectra reveal an exciton binding energy of *E*_b_ = 76.9 meV for 4Br2FBZ-CAB, which is lower than that of BZ-CAB (fig. S13). This value is higher than that of 3D perovskite materials (*44*–*46*) but lower than most 2D layered perovskite materials (*47*–*49*), indicating that the solid solution of metal ions effectively enhances carrier transport. In addition, the PL lifetime of 4Br2FBZ-CAB reaches 13.02 ns, further demonstrating the improved electronic band structure of 4Br2FBZ-CAB (Fig. 6C). As shown in Fig. 6D, the space charge-limited current (SCLC) method is adopted to analyze the trap-state density (*n*_trap_) and charge carrier mobility (μ) of 4Br2FBZ-CAB single crystal. The ohmic region, the trap-filling region, and the Child’s region can be clearly identified as the bias voltage increases. The *n*_trap_ is estimated to be 9.63 × 10^9^ cm^−3^, which is lower than that of 2D perovskites (BA)_2_CsAgBiBr_7_ (4.2 × 10^10^ cm^−3^) (*50*) and (4-amidinopyridine)_2_AgBiBr_8_ (5.74 × 10^10^ cm^−3^) (*51*), much lower than that of the conventional inorganic semiconductor material CdTe (10^13^ to 10^14^ cm^−3^) (*52*). This low defect density may be attributed to the solid solution of metal ions. Moreover, in the Child’s region, the μ was preliminarily estimated to be ∼11.6 cm^2^ V^−1^ s^−1^, comparable to that of 2D lead-based perovskites, confirming efficient charge transport properties of 4Br2FBZ-CAB (*47*, *53*). In general, a large bulk resistivity is associated with low dark current and noise in a device, establishing it as a key parameter for radiation detectors. As depicted in Fig. 6E, 4Br2FBZ-CAB exhibits a large bulk resistivity (ρ) of 1.87 × 10^11^ Ω cm, which is lower than that of BZ-CAB and surpasses most reported perovskite single crystal (SC) radiation detectors (*54*–*57*). Meanwhile, the carrier mobility-lifetime product (μτ) of 4Br2FBZ-CAB is 8.22 × 10^−4^ cm^2^ V^−1^, which is higher than the value of 5.66 × 10^−5^ cm^2^ V^−1^ obtained for BZ-CAB, confirming the significant potential of 4Br2FBZ-CAB for radiation detection (Fig. 6F).

### X-ray detection performance

**Fig. 7.**
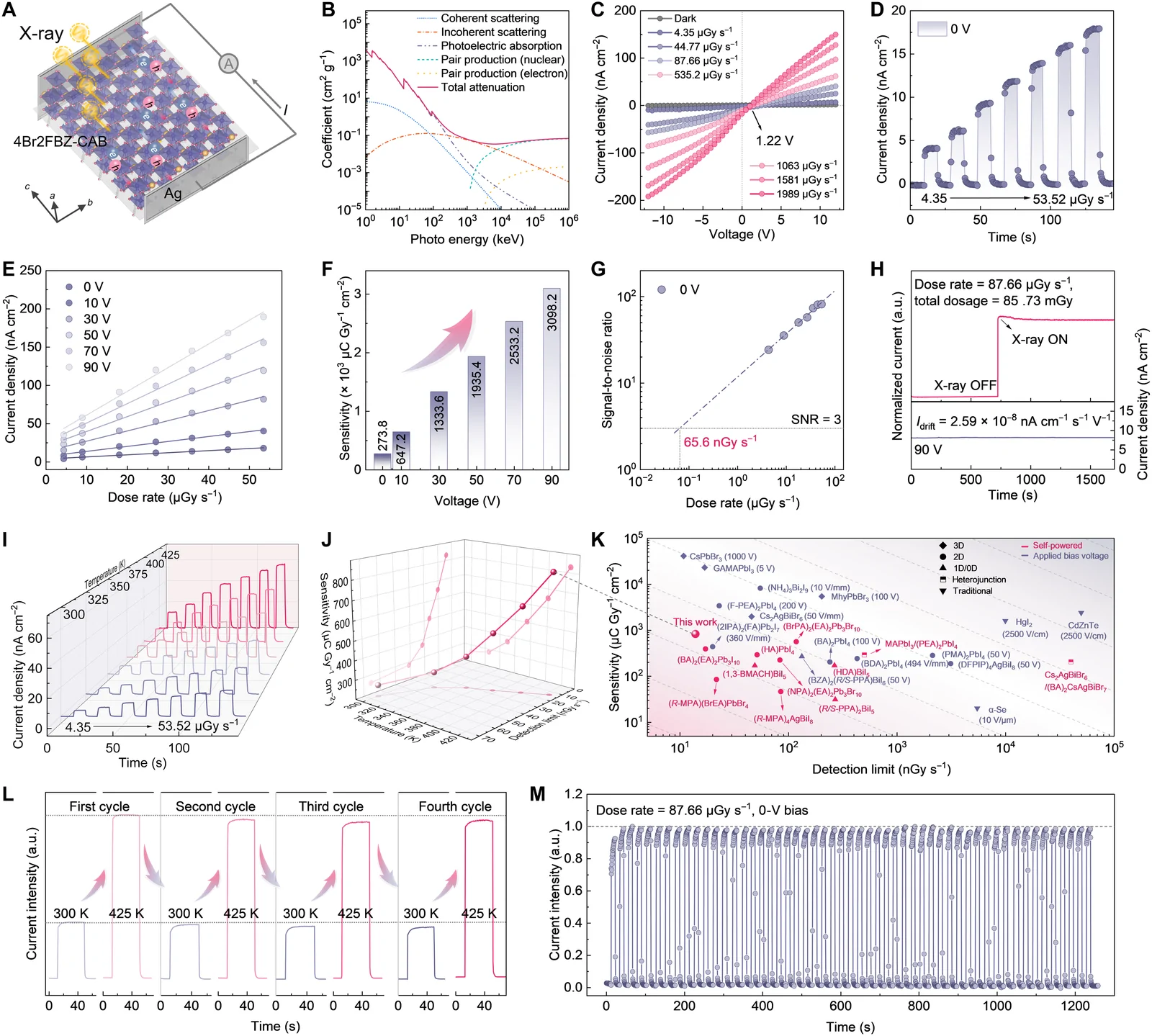
X-ray detection performance. (**A**) Schematic diagram of the 4Br2FBZ-CAB SC detector. (**B**) The attenuation coefficient of various forms of x-ray interaction. (**C**) Current-voltage curves of the detector at irradiation and in the dark along the *c* axis. (**D**) Current density versus time curve of the SC detector at 0-V bias. (**E**) X-ray–generated current density versus dose rate under different bias voltages. (**F**) Sensitivity at various bias voltages. (**G**) Detection limit of the detector at 0-V bias. (**H**) Dark current and response current under 0-V bias and the dark current drift under 90-V bias. (**I**) The on-off photocurrent responses from 4.35 to 53.52 μGy_air_ s^−1^. (**J**) sensitivity and detection limit under the 0-V bias at the temperature from 300 to 425 K. (**K**) Comparison of x-ray detection performance of 4Br2FBZ-CAB and other reported materials: CsPbBr_3_ (*63*), GAMAPbI_3_ (*64*), MhyPbBr_3_ (*59*), Cs_2_AgBiBr_6_ (*65*), (NH_4_)_3_Bi_2_I_9_ (*66*), (F-PEA)_2_PbI_4_ (*67*), (2IPA)_2_(FA)Pb_2_I_7_ (*68*), (BA)_2_PbI_4_ (*69*), (BDA)_2_PbI_4_ (*70*), (PMA)_2_PbI_4_ (*71*), (DFPIP)_4_AgBiI_8_ (*16*), (BZA)_2_(*R/S*-PPA)BiI_6_ (*72*), (BrPA)_2_(EA)_2_Pb_3_Br_10_ (*54*), (BA)_2_(EA)_2_Pb_3_I_10_ (*73*), (NPA)_2_(EA)_2_Pb_3_Br_10_ (*74*), (HA)PbI_4_ (*75*), (*R*-MPA)(BrEA)PbBr_4_ (*56*), (*R*-MPA)_4_AgBiI_8_ (*76*), (*R/S*-PPA)_2_BiI_5_ (*77*), (1,3-BMACH)BiI_5_ (*78*), (HAD)BiI_5_ (*79*), MAPbI_3_/(PEA)_2_PbI_4_ (*60*), Cs_2_AgBiBr_6_/(BA)_2_CsAgBiBr_7_ (*80*), HgI_2_ (*81*), α-Se (*82*), and CdZnTe (*83*). (**L**) The x-ray response current of the detector after thermal cycling. (**M**) On/off stability of the device at 425 K under 0-V bias.

On the basis of the excellent ferroelectric properties of compound 4Br2FBZ-CAB and its optimized charge transport structure, we next investigated the x-ray detection performance, with a schematic diagram of the detector shown in Fig. 7A. First, we analyzed the various forms of 4Br2FBZ-CAB interaction with x-rays. As with most materials, x-ray photons are primarily absorbed via the photoelectric effect in the low-energy region (Fig. 7B). Its absorption coefficient and attenuation efficiency surpass those of traditional semiconductor materials such as Si and α-Se, demonstrating significant potential for x-ray detection (fig. S14). The ferroelectric bulk photovoltaic effect refers to the generation of a stable photocurrent and photovoltage in a ferroelectric crystal device under continuous illumination along its polar axis. This enables spontaneous separation and transport of photogenerated carriers, achieving self-powered detection. As shown in Fig. 7C, current-voltage curves were measured at different x-ray doses, revealing an open-circuit voltage of 1.22 V, which provides the driving force for self-powered x-ray detection. Photocurrent signals of SC devices recorded under x-ray irradiation without an applied bias voltage and various dose rates are shown in Fig. 7D. The device exhibits excellent stability and a high switching ratio under 0-V bias. Furthermore, it demonstrates outstanding detection performance under different bias conditions (fig. S15). At various bias voltages, x-ray current density of the device exhibits a high linear relationship with the x-ray dose rate (Fig. 7E). The device achieved a sensitivity of 273.8 μC Gy_air_^−1^ cm^−2^ at 0-V bias, and the sensitivity reached as high as 3098.2 μC Gy_air_^−1^ cm^−2^ at 90-V bias (Fig. 7F). The high sensitivity value at 0 V bias is markedly higher than the detection sensitivity of traditional devices under high bias voltages (e.g., the sensitivity of α-Se is 20 μC Gy_air_^−1^ cm^−2^ under a high electric field of 10^5^ V cm^−1^) (*58*) and surpasses that of most reported self-powered x-ray detectors (*59*, *60*). Meanwhile, the sensitivity of the BZ-CAB SC detector is lower than that of the 4Br2FBZ-CAB detector, indicating that the solid solution of metal ions facilitates carrier transport and enables greater photoresponse (fig. S16). In addition, the detection limit is another critical parameter for x-ray detectors, defined as the dose rate at which the signal-to-noise ratio (SNR) is equal to 3. The detection limit at 0-V bias was 65.6 nGy_air_ s^−1^, which is lower than the detection limit of commercial α-Se detectors (5500 nGy_air_ s^−1^), demonstrating outstanding detection capability for weak radiation signals (Fig. 7G) (*58*). The operational stability is a key indicator for the practical application of radiation detectors. Figure 7H demonstrates excellent stability under continuous irradiation, with a negligible decay in photocurrent observed after prolonged operation. Meanwhile, the dark current stability of device was measured at 90-V bias, resulting in a calculated baseline drift value of 2.59 × 10^−8^ nA cm^−1^ s^−1^ V^−1^. This value is comparable to that of the recently reported perovskite detector using (BDA)_4_AgBiBr_8_ (1.56 × 10^−8^ nA cm^−1^ s^−1^ V^−1^) (*55*), confirming that organic cations can effectively suppress harmful ion migration.

Thanks to the high *T*_c_ of ferroelectric 4Br2FBZ-CAB, the device can operate under 0-V bias voltage over a wide temperature range, considerably expanding the application fields of radiation detection. We investigated the self-powered detection response at different operating temperatures ranging from 300 to 425 K, as shown in Fig. 7I. It can be clearly observed that as temperature increases, the dark current density remains nearly almost unchanged under 0-V bias, while the photocurrent density gradually improves. Although the lattice vibration scattering induced by increasing temperature may cause a decrease in carrier mobility, the increased temperature activates a higher carrier concentration and extends the effective carrier lifetime, leading to an increase in photocurrent (*61*, *62*). This is evidenced by the increase in the μτ with increasing temperature (fig. S17). The sensitivity of the detector increased from 273.8 μC Gy_air_^−1^ cm^−2^ at 300 K to 896.9 μC Gy_air_^−1^ cm^−2^ at 425 K, accompanied by an improved SNR and a reduced detection limit to 13.9 nGy_air_ s^−1^ (Fig. 7J and fig. S18). We summarized the x-ray detection limits and sensitivity values of some reported materials in Fig. 7K and table S6. The results show that the 4Br2FBZ-CAB device at 0-V bias demonstrates superiority in sensitivity and detection limits compared to other reported self-powered devices and even bias-powered devices, particularly when compared to traditional materials. Furthermore, following thermal treatment at 425 K for 48 hours, no extra diffraction peaks are observed in the PXRD pattern of 4Br2FBZ-CAB, demonstrating superior phase stability (fig. S19). The operational stability of the device was assessed at a high temperature of 425 K. As illustrated in Fig. 7L, the device retains excellent self-powered response after four thermal cycles, exhibiting only minor degradation. Moreover, the device exhibits outstanding stability during repeated switching operations at 425 K under a dose rate of 87.66 μGy_air_ s^−1^ (Fig. 7M). In addition, under sustained irradiation, the device maintains stability after 600 s, showing only a 4.2% attenuation relative to the initial photoresponse (fig. S20). Thus, the device not only exhibits excellent x-ray detection performance at high temperature but also demonstrates impressive stability, providing insights for the development of efficient high-temperature self-powered x-ray detectors.

## DISCUSSION

In summary, we present a high-*T*_c_ heterovalent metal solid solution double perovskite ferroelectric, (4Br2FBZ)_2_CsAgBiBr_7_ (4Br2FBZ-CAB, 4Br2FBZ = 4-bromo-2-fluorobenzylammonium), through a strategy of cation-engineered symmetry regulation. The tailoring of organic cations drives the transition of structural symmetry inducing the formation of a heterovalent metal solid solution. The metal ions Ag and Bi changed from an alternating arrangement to a mixed-site solid solution pattern, effectively optimizing the electronic band structure to enhance carrier transport. Meanwhile, 4Br2FBZ-CAB exhibits a high *T*_c_ of 484 K, significantly expanding the family of high-*T*_c_ lead-free ferroelectrics. Benefiting from its remarkable photoelectric and ferroelectric properties, a high self-powered x-ray detection sensitivity of 273.8 μC Gy_air_^−1^ cm^−2^ was achieved at room temperature. In addition, this exceptionally high *T*_c_ enables the detector to operate self-powered at high temperatures, resulting in an excellent sensitivity of 896.9 μC Gy_air_^−1^ cm^−2^ and a low detection limit of 13.9 nGy_air_ s^−1^ at 425 K. This finding opens an avenue for expanding the family of high-*T*_c_ lead-free perovskite ferroelectrics with superior semiconducting properties, enabling efficient photodetection.

## MATERIALS AND METHODS

### Materials

The following chemicals were used: cesium carbonate (Cs_2_CO_3_, 99.9%, Aladdin), silver oxide (Ag_2_O, 99.7%, Aladdin), bismuth oxide (Bi_2_O_3_, 99.9%, Aladdin), 4-bromo-2fluorobenzylammonium (4Br2FBZ, 98%, Aladdin), 4-bromobenzylammonium (4BrBZ, 98%, Aladdin), benzylammonium (BZ, 99%, Aladdin), n-butylammonium (BA, 99%, Aladdin), n-propylamine (PA, 99.5%, Aladdin), and hydrobromic acid (HBr, 40%, Aladdin). All the chemicals were commercially available and used without any further purification.

### Synthesis and crystal growth

All compounds were synthesized by combining a stoichiometric ratio of organic cations, Ag_2_O, Bi_2_O_3_, and Cs_2_CO_3_ in a concentrated aqueous HBr solution. This solution was heated with continuous stirring at 373 K until the initially produced orange solid was fully dissolved. Their microcrystalline powders were obtained by slowly cooling to room temperature. The high-quality bulk single crystals of 4Br2FBZ-CAB are grown in a closed heating oven through the temperature-lowering method from a precursor solution from 353 to 298 K.

### Single crystal and PXRD

A Bruker D8 Quser/Venture diffractometer with Mo Kα radiation (λ = 0.77 Å) is used to analyze the single-crystal structure of 4Br2FBZ-CAB. The crystal structure was solved by the direct method and refined by the full matrix method based on *F*^2^ using the SHELXTL program. Powder XRD patterns were measured on a Rigaku Miniflex 600 x-ray diffractometer equipped with Cu-Kα in the 2θ range of 5° to 40° with a step width of 0.02°.

### Characterization

The interlayer interactions are analyzed using the CrystalExplorer program. A Netzsch STA 449F3 Jupiter thermal analyzer is used for the thermogravimetric measurement of 4Br2FBZ-CAB under the *N*_2_ flow from room temperature to 1298 K at a heating rate of 15 K min^−1^. DSC measurement was performed on the NETZSCH DSC 200 F3 in the temperature range of 273 to 500 K. The crystalline samples were placed in aluminum crucibles that were heated and cooled with a rate of 10 K/min under the nitrogen atmosphere. The single-crystal devices for dielectric measurements were fabricated by depositing two symmetric Ag electrodes on the two opposite sides along the polar crystallographic *c*-axis direction. Temperature-dependent dielectric constant measurements were performed on an Impedance Analyzer (TongHui TH2828A). The SHG properties were investigated on crystal powered samples with a homemade Q-switched Nd:YVO_4_ laser at 1340 nm (full width at half maximum of 100 fs, repetition rate of 1 kHz). The XPS spectrum measurements were performed on a Thermo Fisher Scientific ESCALAB 250Xi using Al Kα (1486.6 eV). EDS element mapping was obtained by a TALOS F200X G2 TEM. The UV-vis absorption spectrum of 4Br2FBZ-CAB powder is measured on a PerkinElmer Lambda 950 UV-vis-NIR spectrometer with BaSO_4_ as a reference. The PL excitation and emission spectra and temperature-dependent PL spectra of 4Br2FBZ-CAB were recorded on a steady-state fluorescence spectrometer (FLS1000, Edinburgh Instrument). The decay curve was obtained using the kinetic mode of FLS1000 with a 450-nm laser diode as the excitation source. The PFM measurement was carried out on a commercial piezoresponse force microscope (Oxfordinstrument, Cypher ES) with high-voltage package. PFM is based on the atomic force microscopy, with an ac drive voltage applied to the conductive tip. ConductiveTi/Ir-coated silicon probes (ASYELEC.01-R2, Oxford instrument) were used for domain imaging and polarization switching studies, with a nominal spring constant of ∼2.8 nN/nm and a free-air resonance frequency of ∼75 kHz.

### X-ray detection

The Ag/4Br2FBZ-CAB single-crystal/Ag devices were fabricated along the crystallographic *c*-axis orientation, with an effective area of 0.0739 mm^2^ and a detector thickness of 0.37 mm. X-rays were incident from the top of the device and effectively absorbed by the single crystal. The current-voltage (*I-V*) traces and current-time (*I-t*) curves of 4Br2FBZ-CAB device are recorded by a Keithley 6517B high-precision electrometer. An Amptek Mini-X2 x-ray tube with a silver target (maximum power of 4 W) was used as the x-ray source. The maximum x-ray photons energy is 50 keV, and the peak intensity is at 22 keV. The dose rate of x-ray tube was modulated by changing its tube current and measured by a Radcal Accu-Gold x-ray dosimeter attached with the 10X6-180 ion chamber in an integrating mode.
