# Supplementary material for: Molecular-driven lead-free halide photoferroelectric solid solution for high-temperature sensitive self-powered x-ray detection
Source: Sci Adv. 2026 Aug 21;12(34):eaef7693. doi: 10.1126/sciadv.aef7693 (PMC13496202; doi:10.1126/sciadv.aef7693)
Supplement: Supplementary file 1 [file sciadv.aef7693_sm.pdf]

Science Advances
AAAS

Supplementary Materials for

Molecular-driven lead-free halide photoferroelectric solid solution for high-temperature sensitive self-powered x-ray detection

Lijun Xu *et al.*

Corresponding author: Junhua Luo, jhluo@fjirsm.ac.cn

Sci. Adv. **12**, eaef7693 (2026)
DOI: 10.1126/sciadv.aef7693

The PDF file includes:

Supplementary Text
Figs. S1 to S20
Tables S1 to S6
Legend for data S1

Other Supplementary Material for this manuscript includes the following:

Data S1

Supplementary Text

Calculation of exciton binding energy (E_b). Fitting the PL intensity as a function of temperature could be used to determine the exciton binding energy (E_b) according to the equation shown below,

$$I_T = \frac{I_0}{1 + Ae^{-E_b/k_B T}} \quad (1)$$

where I_T represents the integrated PL intensity at the measured temperature T, I_0 is the PL intensity at 80 K, k_B is the Boltzmann constant, and A is the fitting parameter.

Calculations of trap-state density (n_{trap}) and charge carrier mobility (μ). The n_{trap} is calculated by the equation,

$$n_{trap} = \frac{2\varepsilon\varepsilon_0 V_T}{qL^2} \quad (2)$$

where V_T is the threshold voltage, L is thickness, q denotes the electron charge, ε and ε_0 denote the relative and vacuum dielectric constant, respectively.

The μ was calculated according to Mott–Gurney's power Law,

$$\mu = \frac{8JL^3}{9\varepsilon\varepsilon_0 V^2} \quad (3)$$

where V denotes the applied voltage, L is thickness, and J is the dark current density.

Calculation of carrier mobility-lifetime product ($\mu\tau$). The $\mu\tau$ is key metric for evaluating the semiconductor performance of X-ray detectors, which can be obtained from the modified Hecht equation:

$$I = \frac{I_0 \mu\tau V}{L^2}\left[1 - \exp\left(-\frac{L^2}{\mu\tau V}\right)\right] \quad (4)$$

where I_0, V and L are the saturated response current, the applied bias, and the thickness, respectively.

Calculations of Sensitivity (S) and Signal-to-Noise Ratio (SNR). S is defined as the collected charge per unit area under X-ray irradiation and can be determined by,

$$S = \frac{(J_{\text{ph}} - J_{\text{d}})}{D} \quad (5)$$

where J_{ph} and J_{d} are the current densities under X-ray illumination and in the dark, respectively, and D is the X-ray dose rate.

SNR is calculated by,

$$SNR = \frac{I_{\text{signal}}}{I_{\text{noise}}} \quad (6)$$

$$I_{\text{signal}} = \overline{I_{\text{ph}}} - \overline{I_{\text{d}}} \quad (7)$$

$$I_{\mathrm{noise}} = \sqrt{\frac{1}{\mathrm{n}}\sum_{1}^{\mathrm{n}}(I_{\mathrm{i}} - \overline{I_{\mathrm{ph}}})^2} \quad (8)$$

where I_{signal} is the signal current and is derived by subtracting the average photocurrent (I_{ph}) by the average dark current (I_{d}), and I_{noise} is the noise current and is obtained by calculating the standard deviation of the I_{ph}.

Calculation of dark current drift (I_{drift}). The dark current drift was determined by the following equation,

$$I_{\mathrm{drift}} = (I_{\mathrm{t}} - I_0)/(\mathrm{E} \times \mathrm{S} \times \mathrm{t}) \quad (9)$$

where I_0 is the dark current immediately after stabilization, I_t is the dark current at time t, E is the electric field, S is the device area.

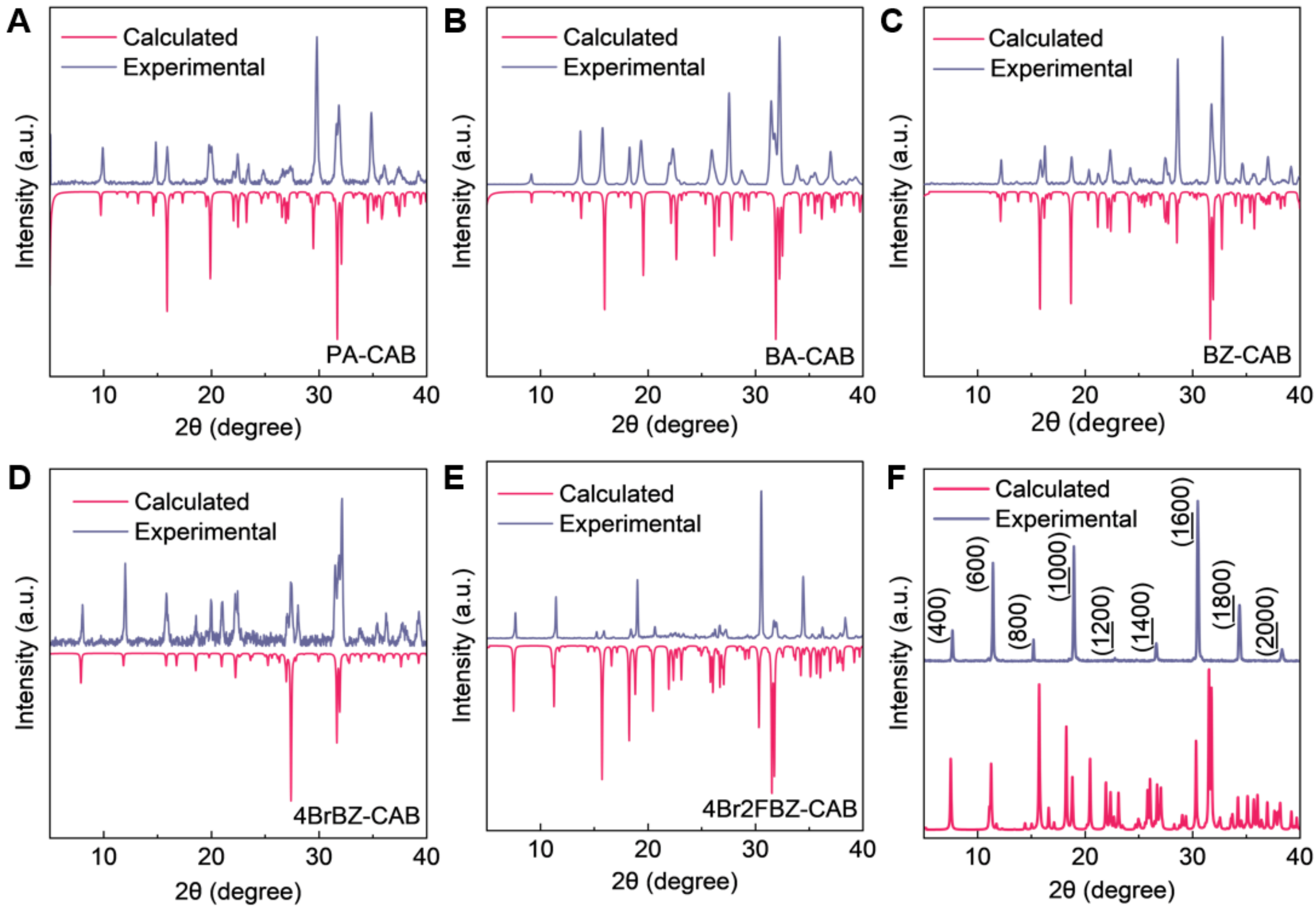

Fig. S1. Calculated and experimental PXRD patterns of PA-CAB (**A**), BA-CAB (**B**), BZ-CAB (**C**), 4BrBZ-CAB (**D**) and 4Br2FBZ-CAB (**E**) measured at room temperature. (**F**) X-ray diffraction patterns of the 4Br2FBZ-CAB single-crystal thin film.

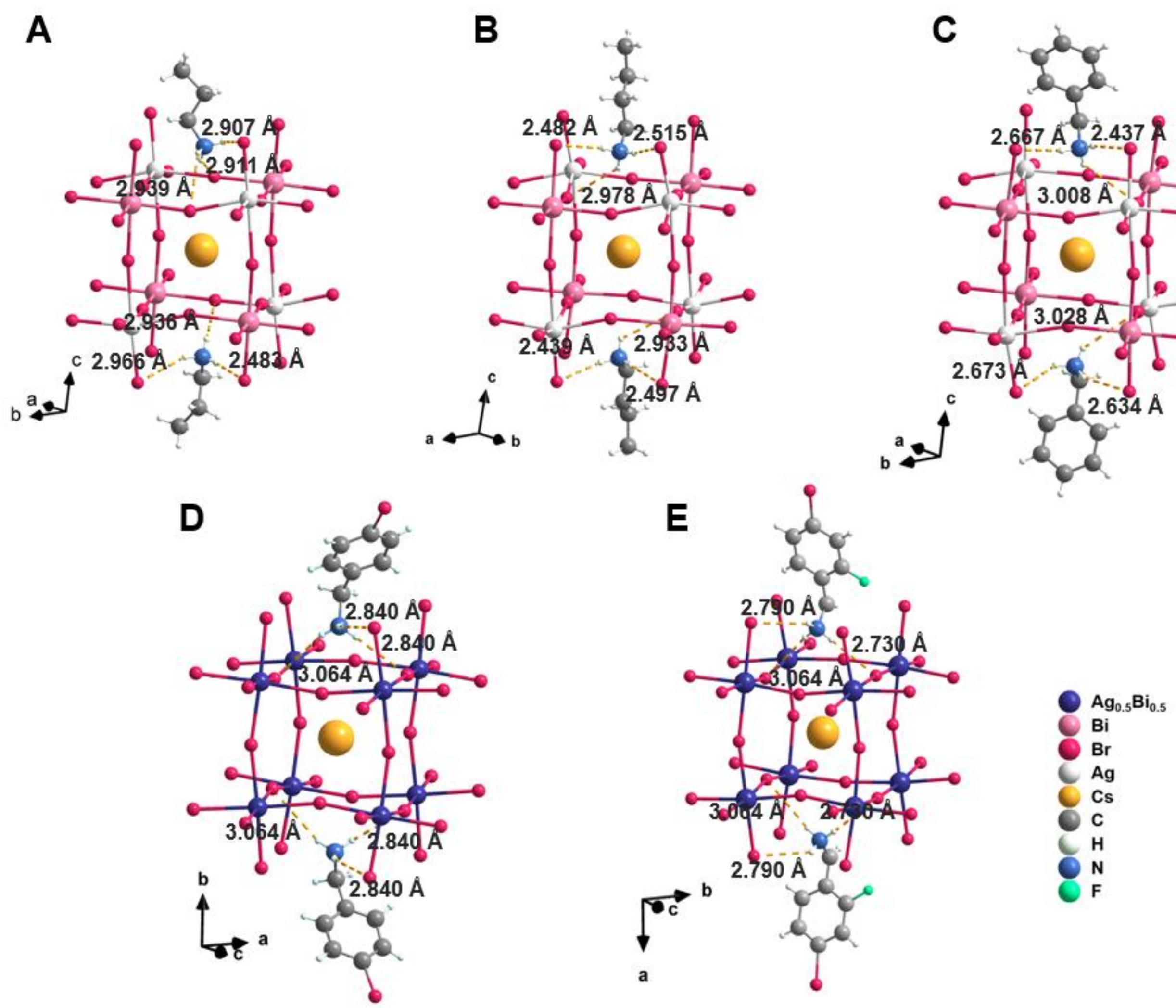

Fig. S2. The inorganic perovskite layers are connected with organic cation layers by N-H···Br hydrogen bonds.

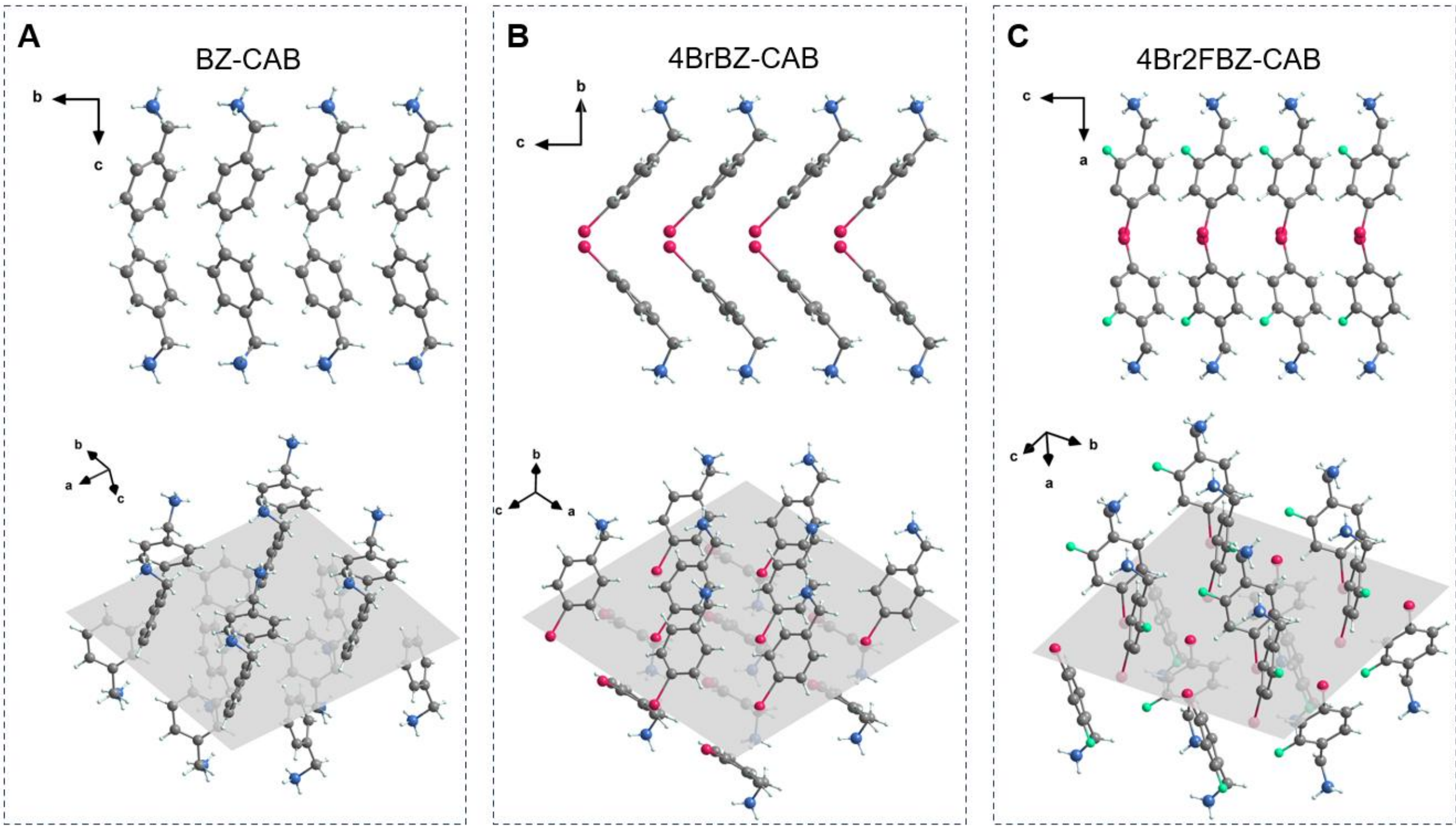

Fig. S3. Organic cation arrangement in BZ-CAB (**A**), 4BrBZ-CAB (**B**), and 4Br2FBZ-CAB (**C**).

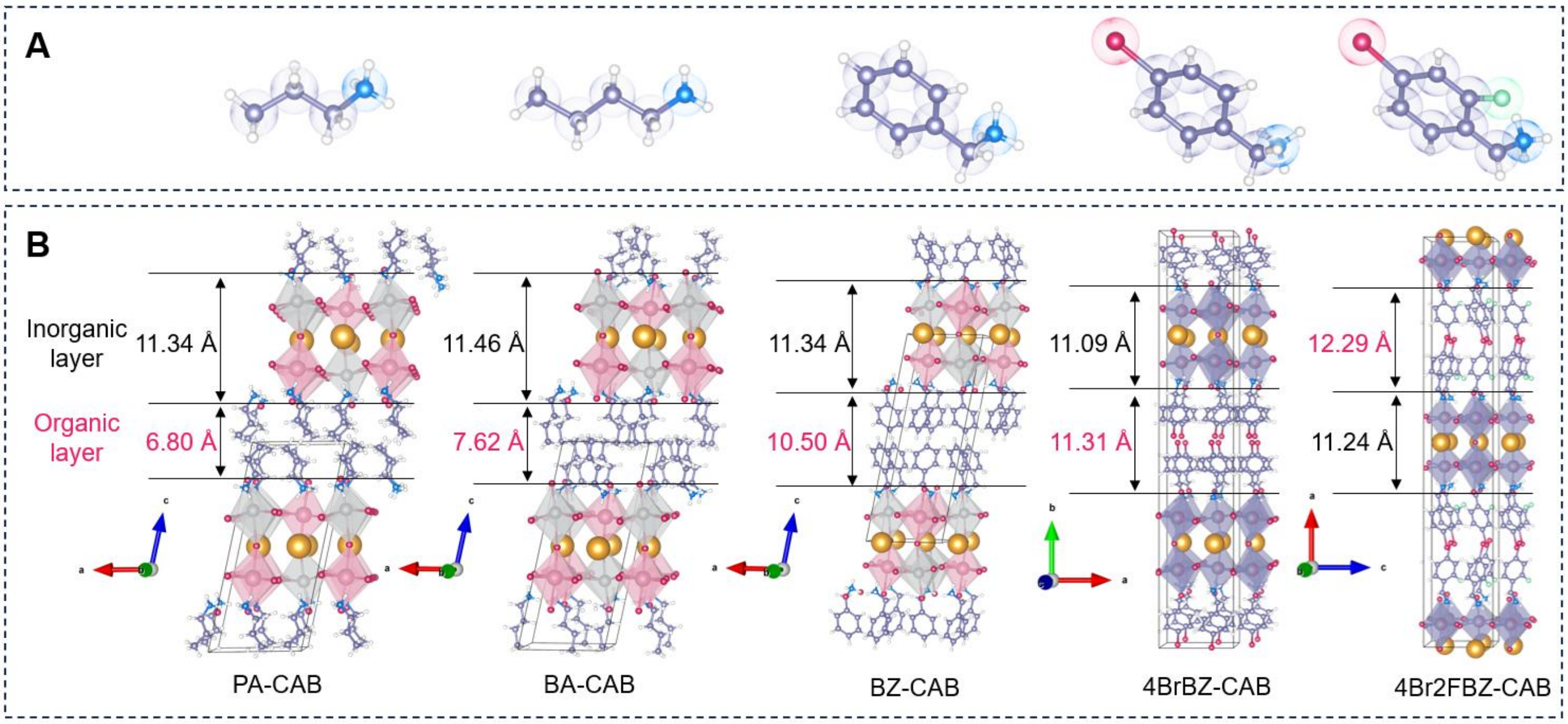

Fig. S4. (**A**) PA, BA, BZ, 4BrBZ, and 4Br2FBZ cations. (**B**) Crystal structures of PA-CAB, BA-CAB, BZ-CAB, 4BrBZ-CAB, and 4Br2FBZ-CAB.

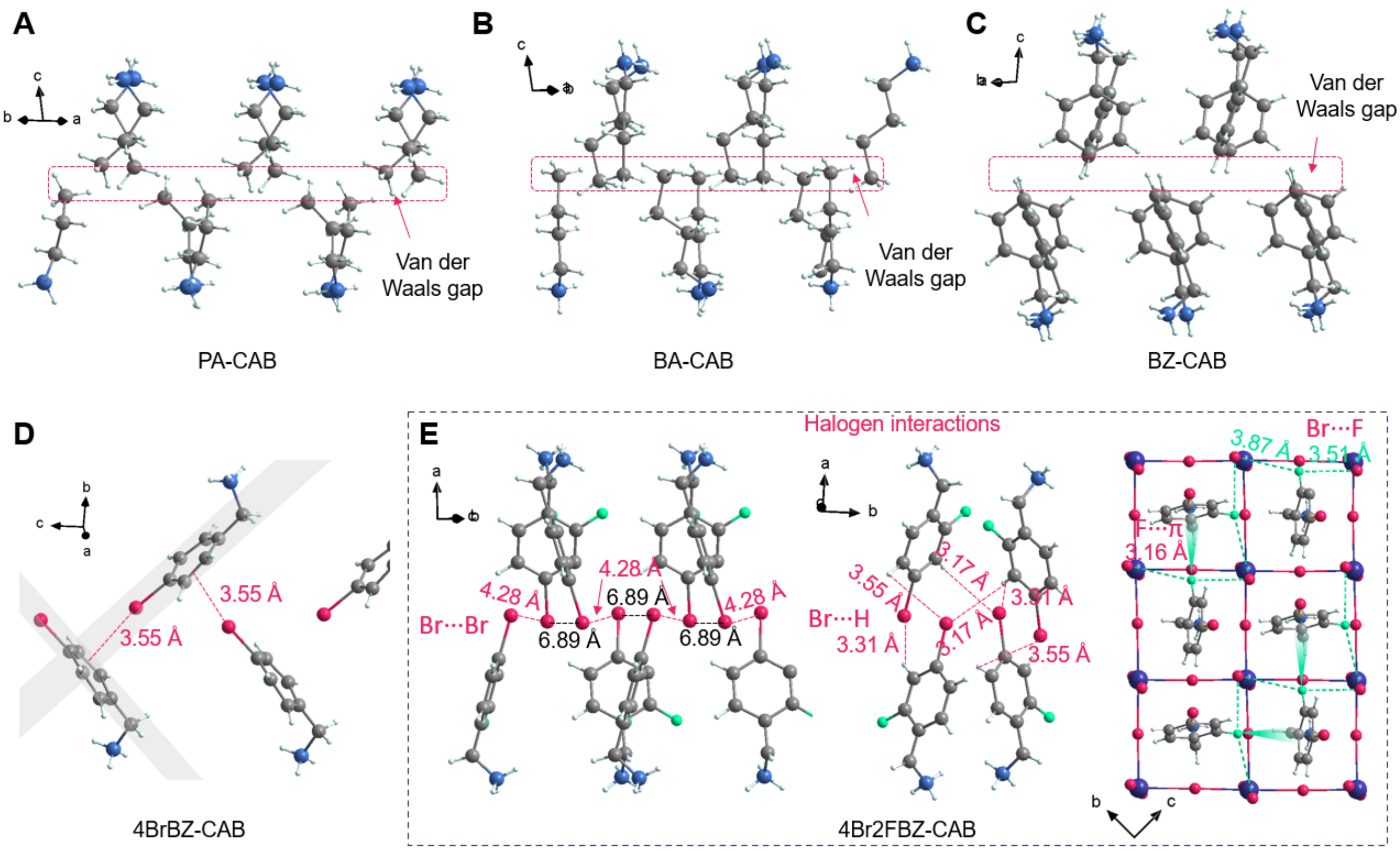

Fig. S5. There is a van der Waals gap between the organic layers of PA-CAB (**A**), BA-CAB (**B**), and BZ-CAB (**C**). (**D**) Br···π interactions between organic layers in 4BrBZ-CAB. (**E**) Interactions between organic layers in 4Br2FBZ-CAB.

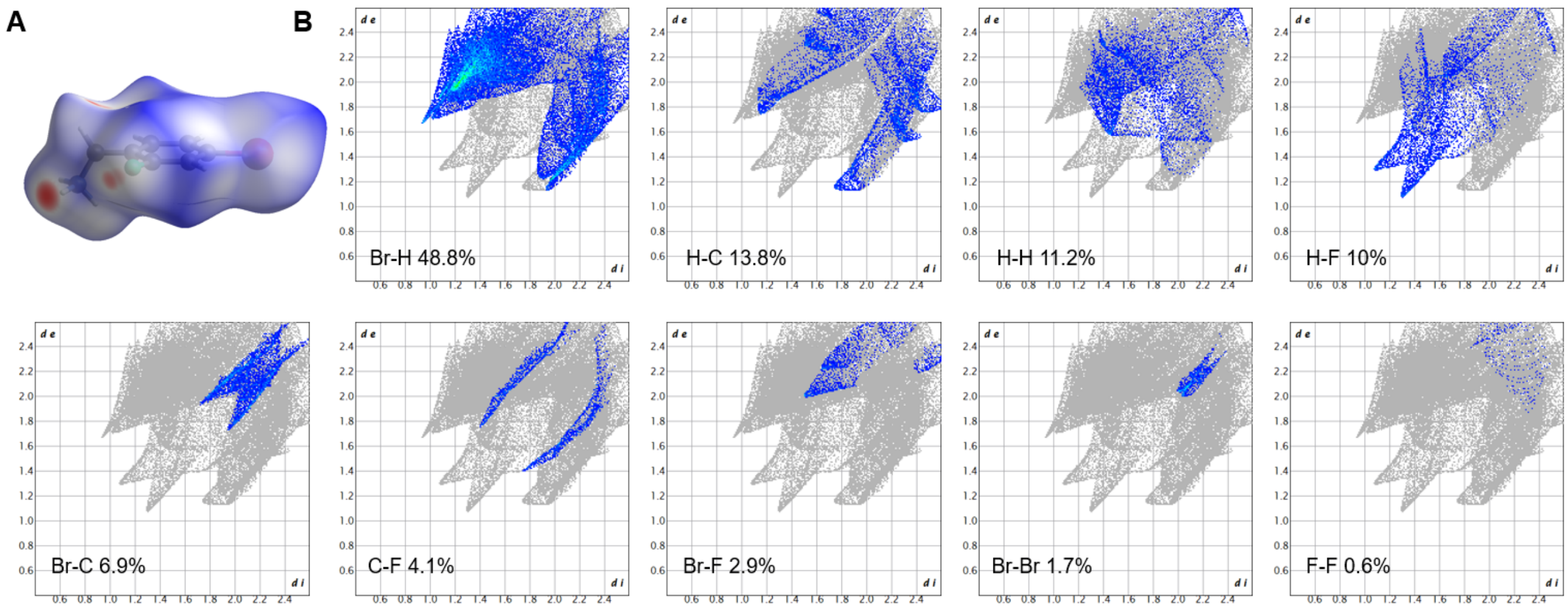

Fig. S6. Hirshfeld surface analysis of the organic cation 4Br2FBZ reveals intermolecular interactions.

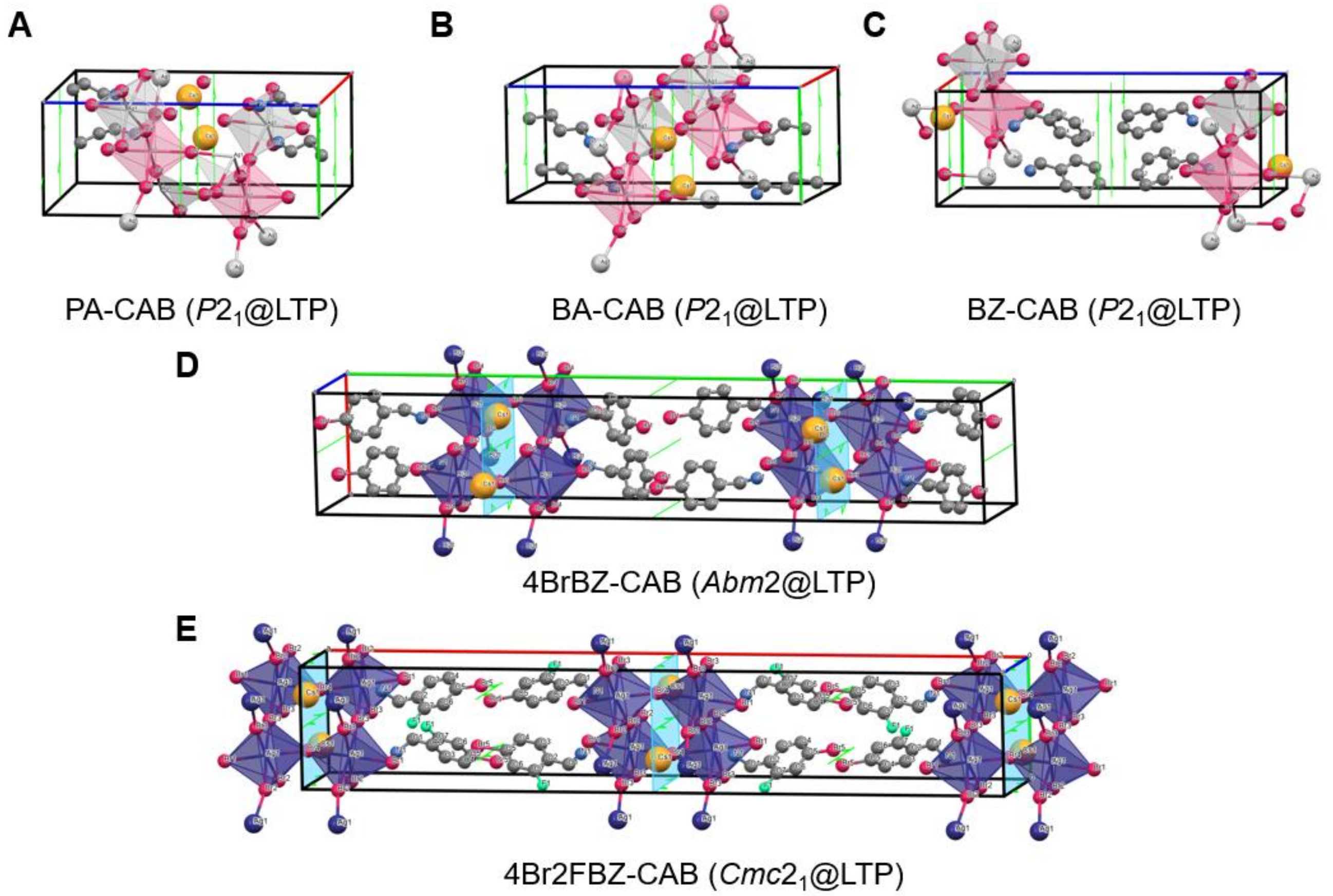

Fig. S7. The symmetry elements of compounds in the low-temperature phase (LTP). For compounds PA-CAB, BA-CAB, and BZ-CAB, the only symmetry element is the 2_1-screw axis along the crystallographic *b*-axis direction. For compound 4BrBZ-CAB and 4Br2FBZ-CAB in addition to the 2_1-screw axis along the crystallographic *c*-axis direction, an *m*-mirror plane exists parallel to the inorganic framework direction.

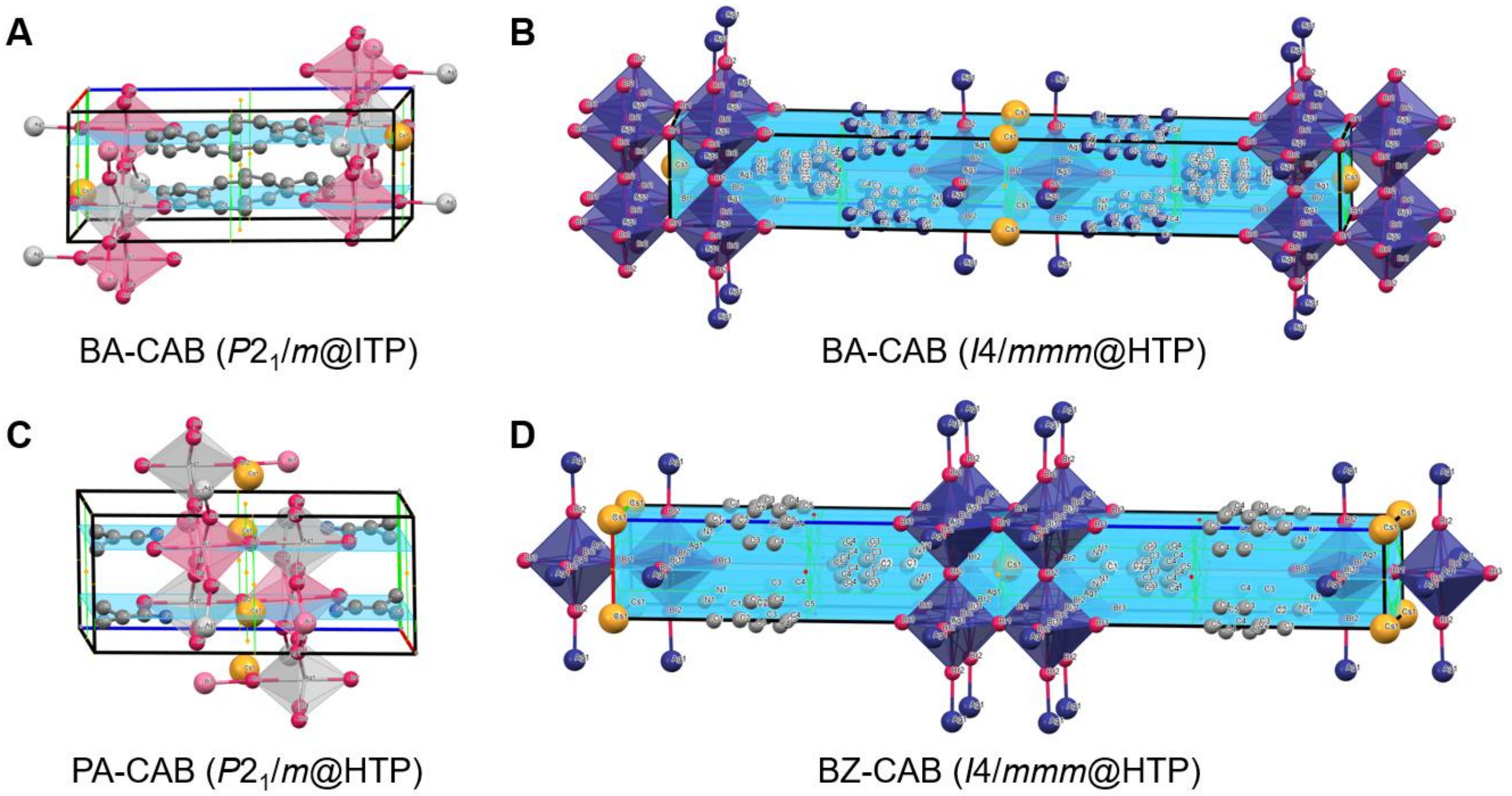

Fig. S8. The symmetry elements of compounds in the intermediate-temperature phase (ITP) or high-temperature phase (HTP). As the temperature increases, PA-CAB and BA-CAB crystallize in the monoclinic system with space group $P2_1/c$, with symmetry elements comprising the 2_1-screw axis along the *b*-axis and the *m*-mirror plane parallel to the *ac* plane. Furthermore, Ag and Bi ions remain in split-sites. The HTP of BA-CAB and BZ-CAB adopt the tetragonal space group *I*4/*mmm* with enhanced symmetry, where Ag and Bi ions occupy mixed-sites.

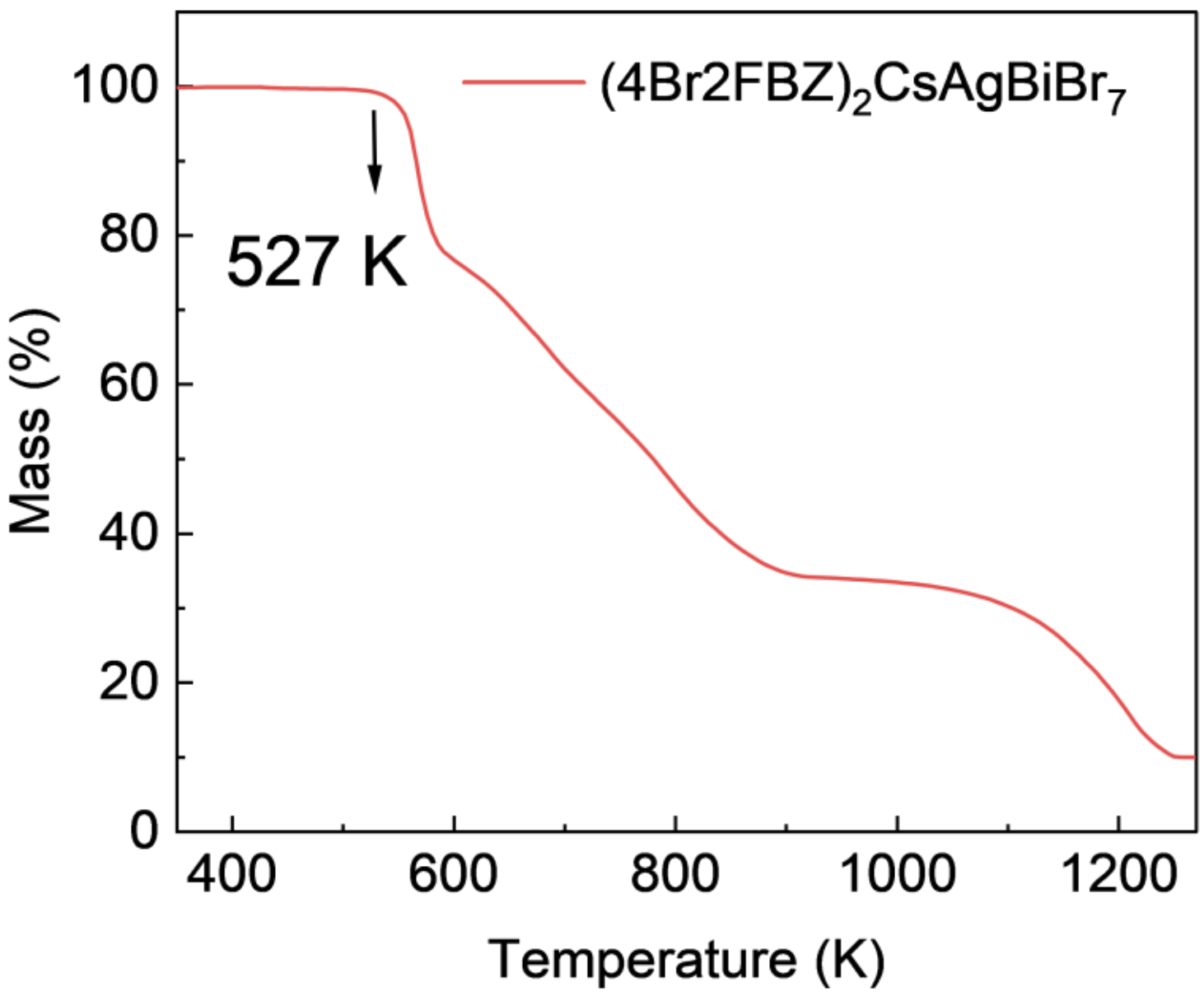

Fig. S9. TG curve of single crystals of 4Br2FBZ-CAB.

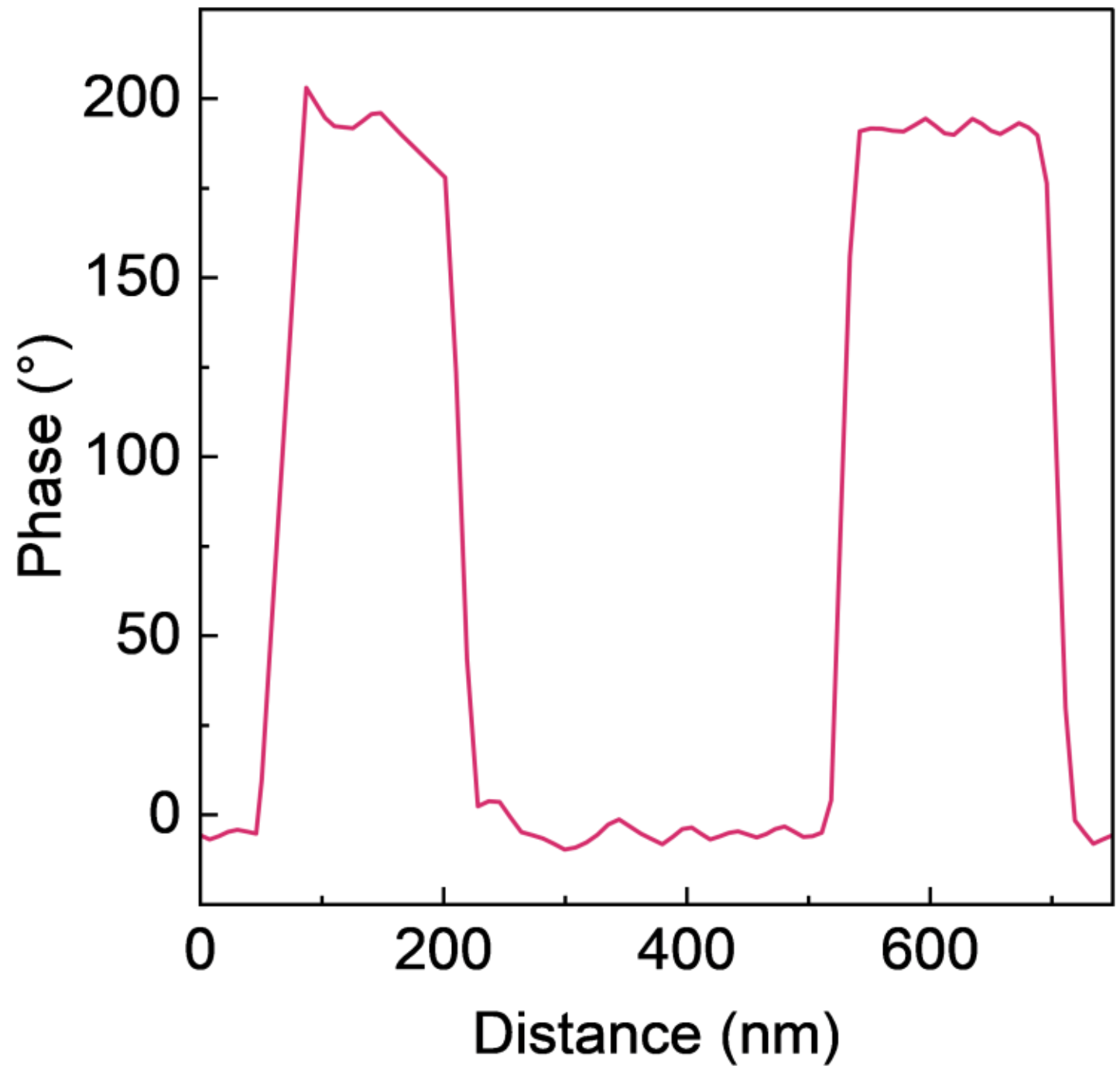

Fig. S10. Phase profile from the PFM phase image.

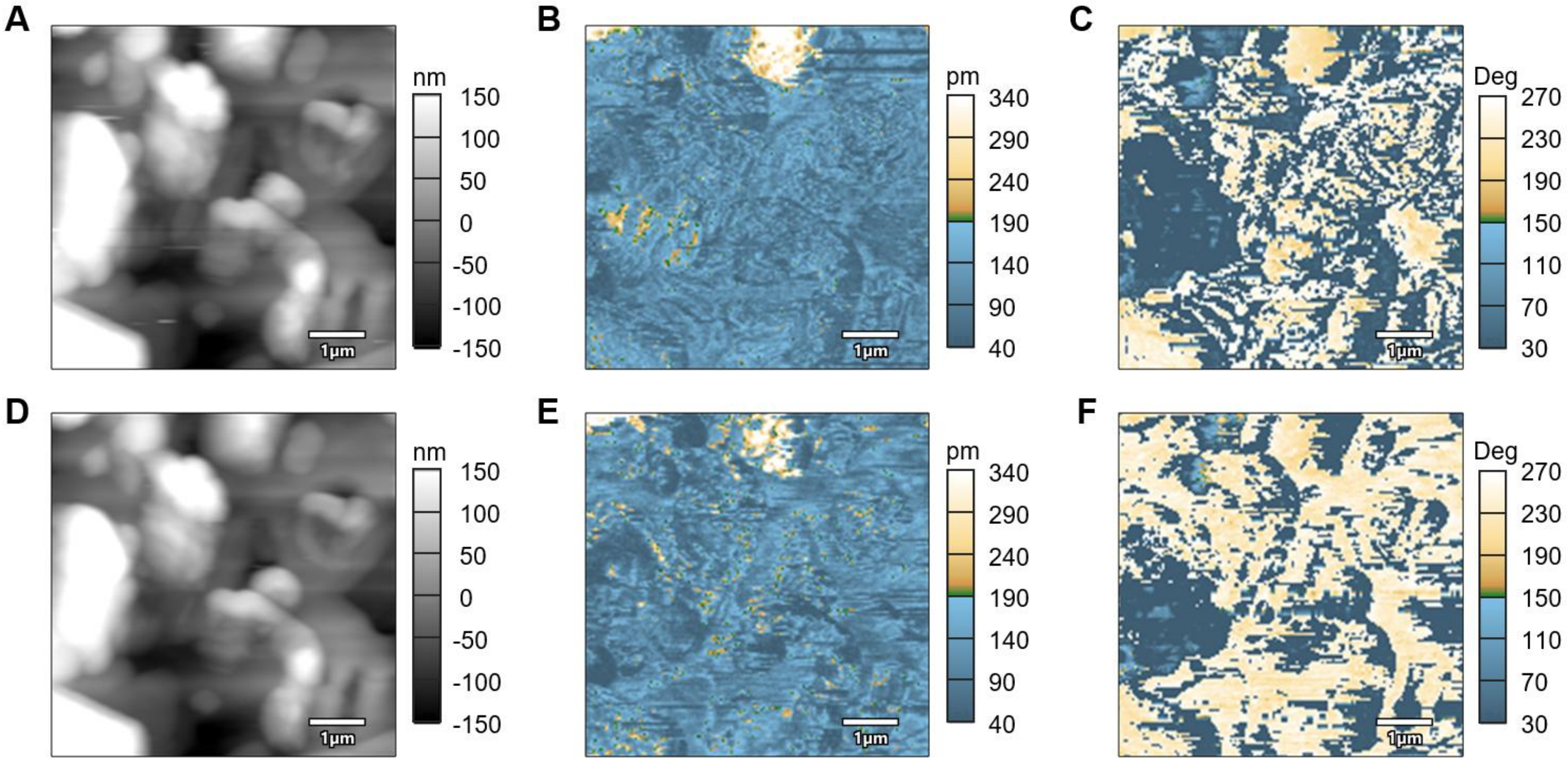

Fig. S11. Topographic images (**A**), PFM amplitude signals (**B**) and PFM phase image (**C**) of the sample surface for the initial state. Topographic images (**D**), PFM amplitude signals (**E**) and PFM phase image (**F**) after the switching with bias at 100 V.

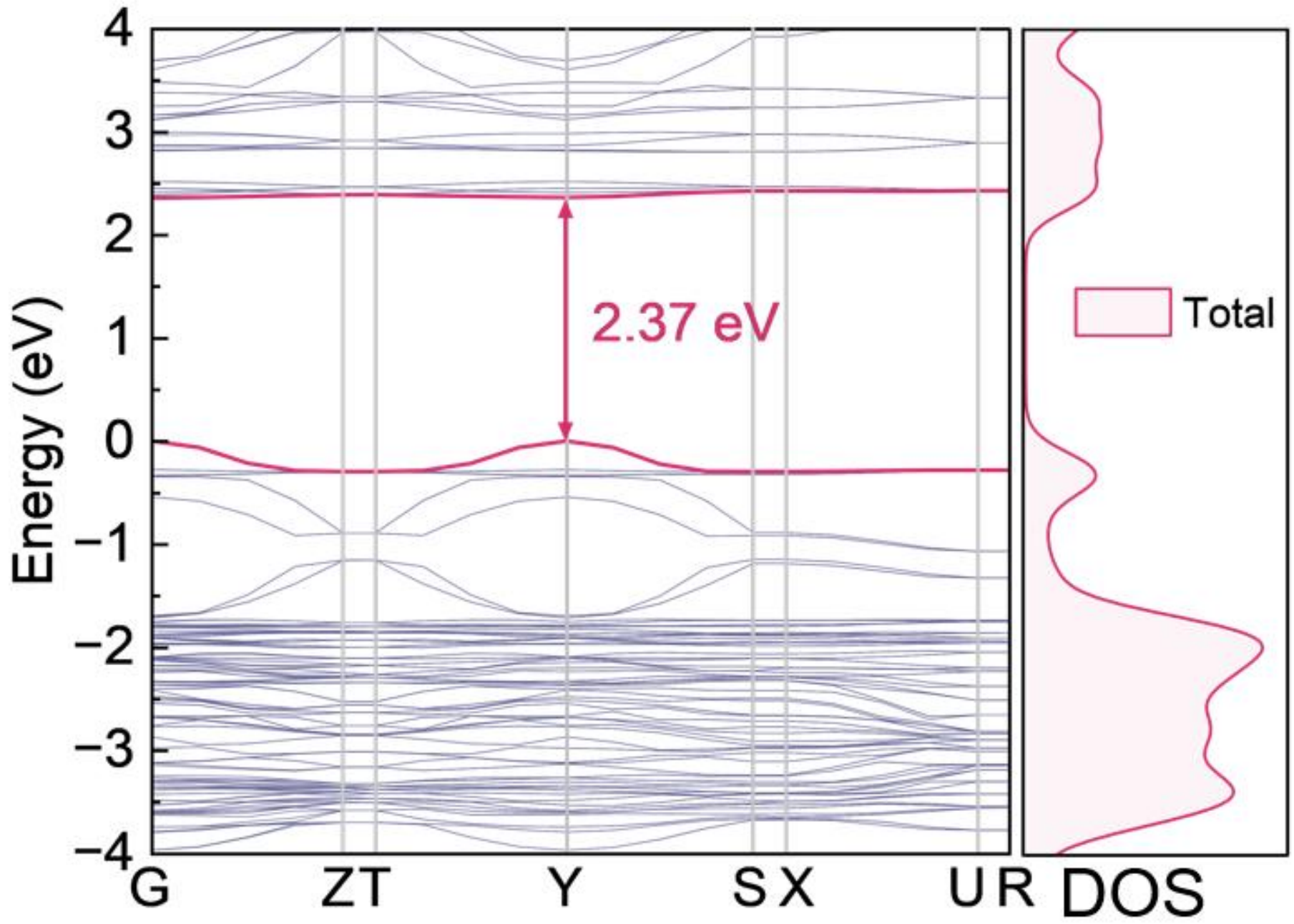

Fig. S12. Calculated bandgap structures and partial density of states (PDOS) of 4Br2FBZ-CAB.

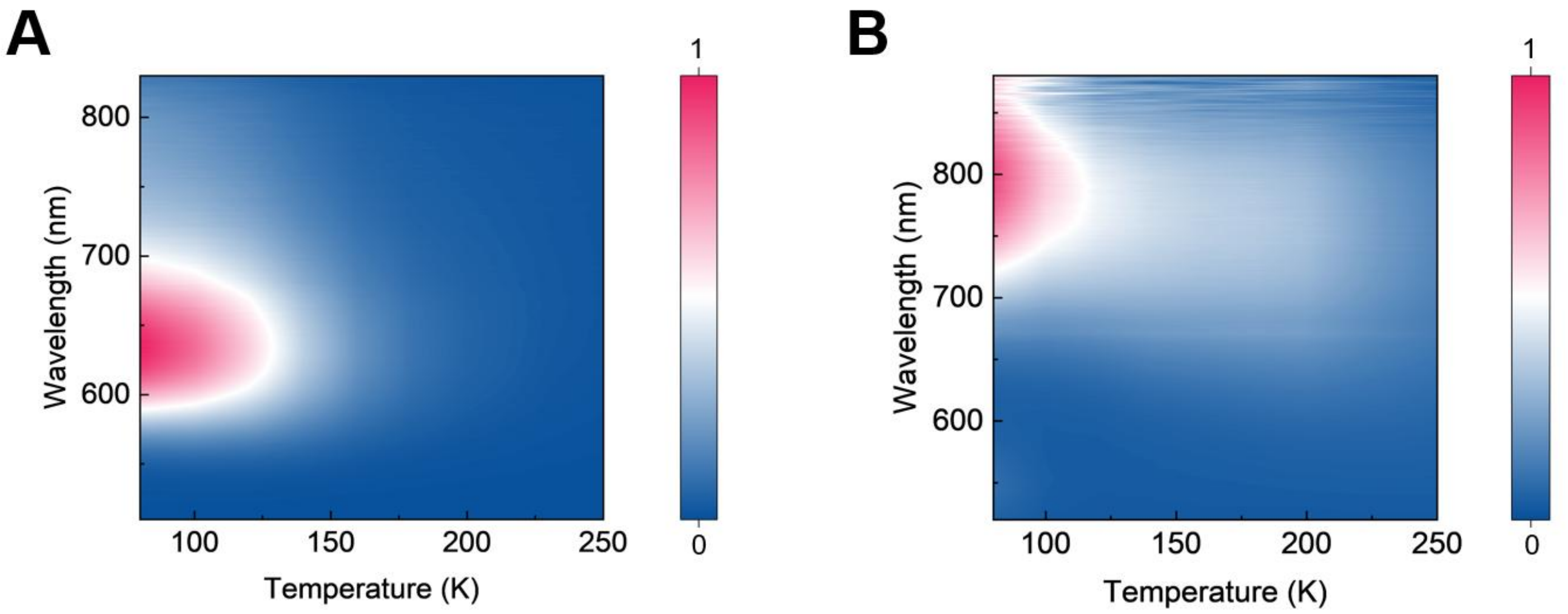

Fig. S13. Temperature-dependent PL emission spectra of 4Br2FBZ-CAB (**A**) and BZ-CAB (**B**).

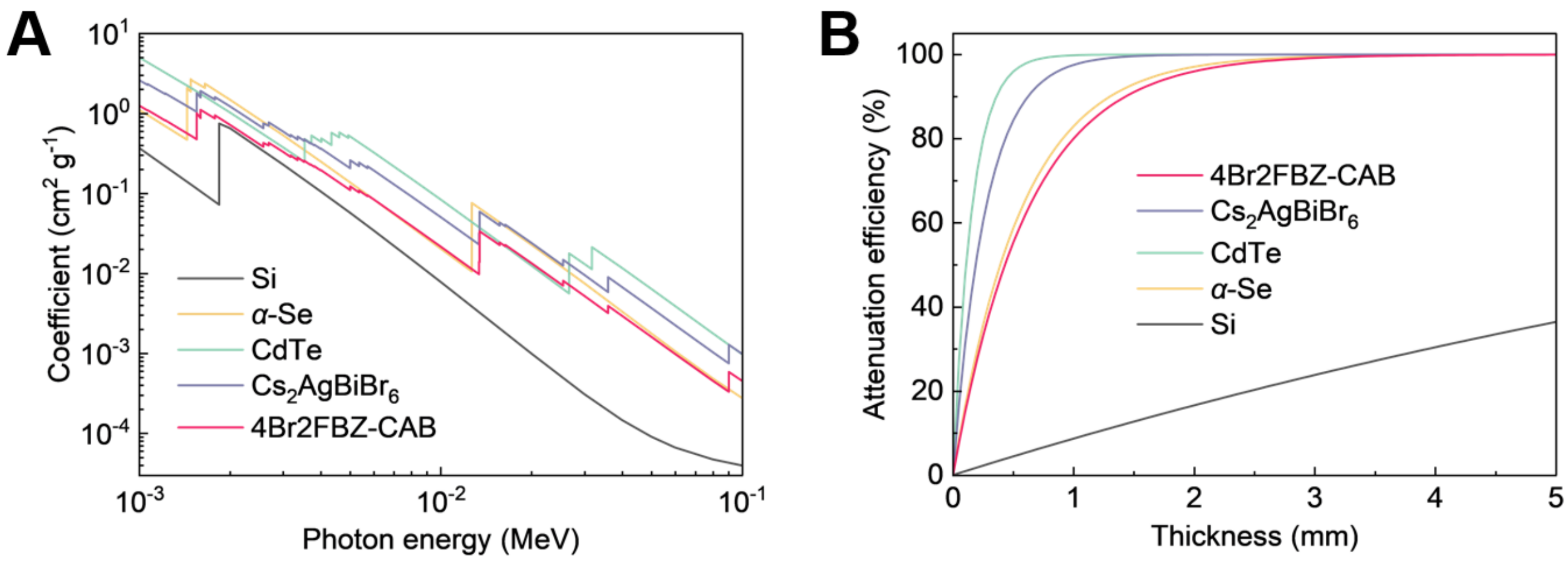

Fig. S14. The attenuation coefficients (**A**) and attenuation efficiencies (**B**) of 4Br2FBZ-CAB and other semiconductors.

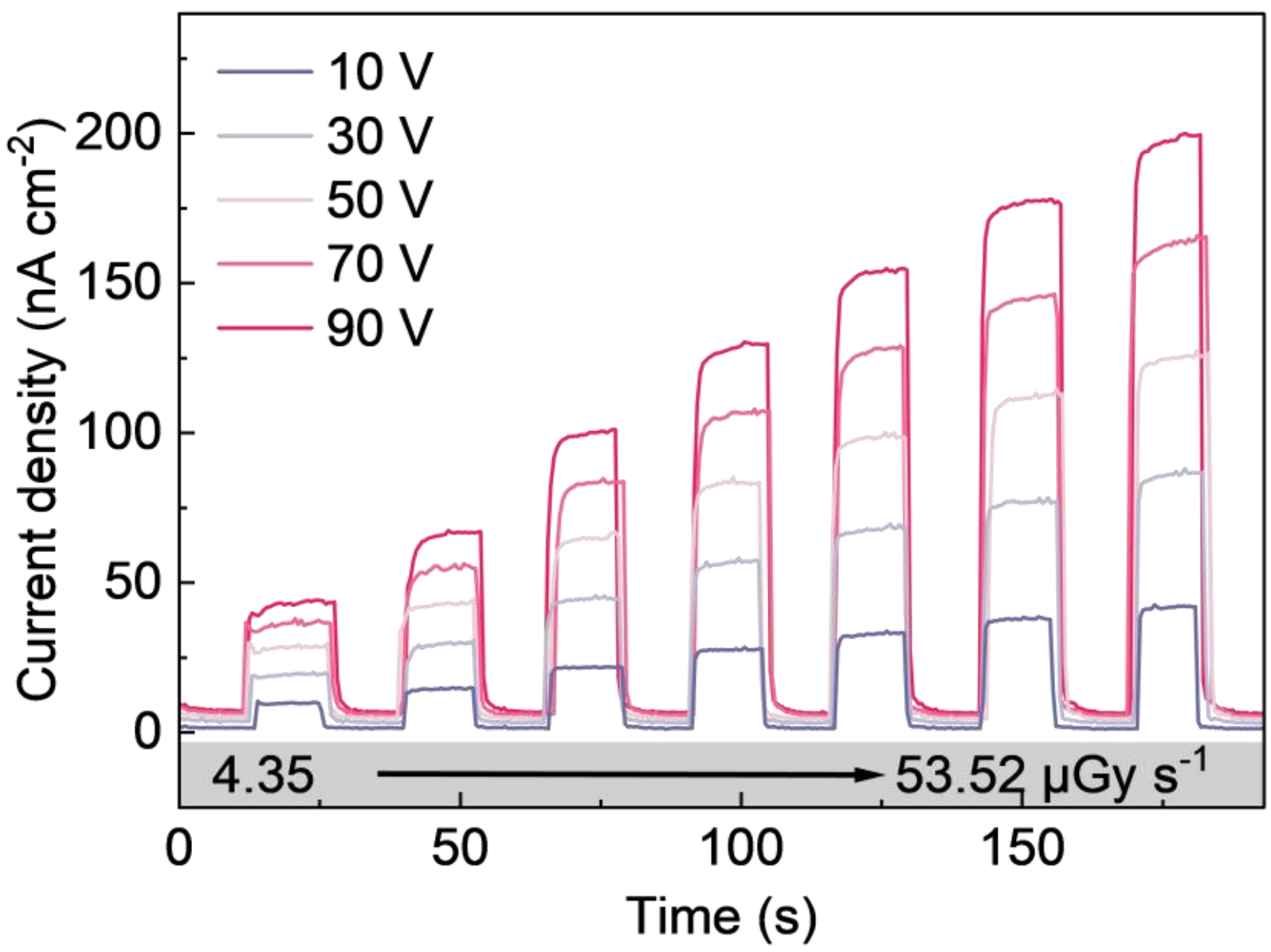

Fig. S15. Current density versus time curves of the 4Br2FBZ-CAB SC detector at different bias voltages.

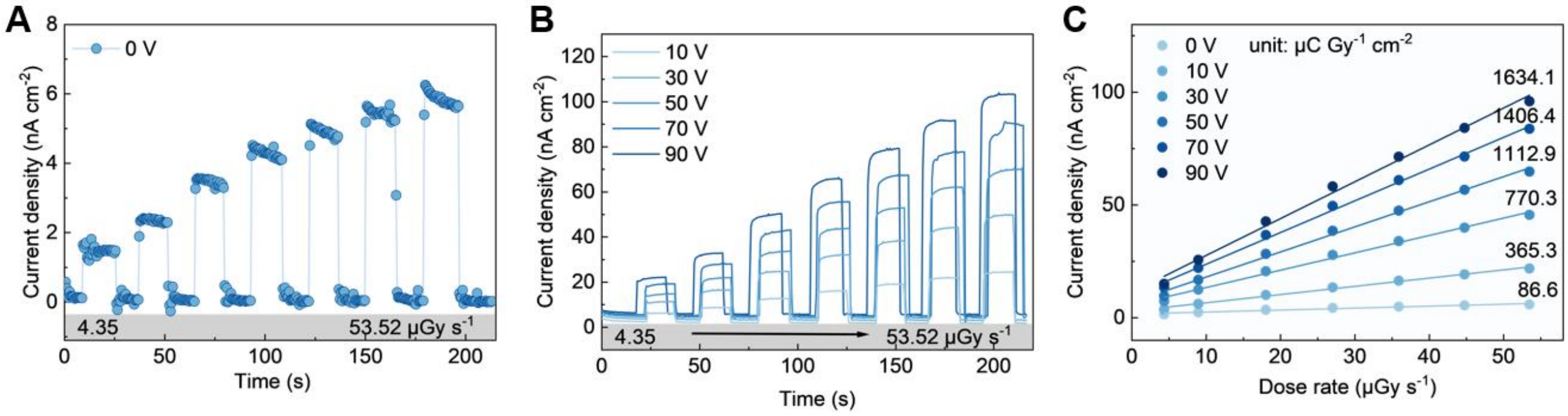

Fig. S16. (**A**) Current density versus time curve of the BZ-CAB detector at 0 V bias. (**B**) Current density versus time curves of the BZ-CAB detector at different bias voltages. (**C**) The sensitivity of BZ-CAB detector at different bias voltages.

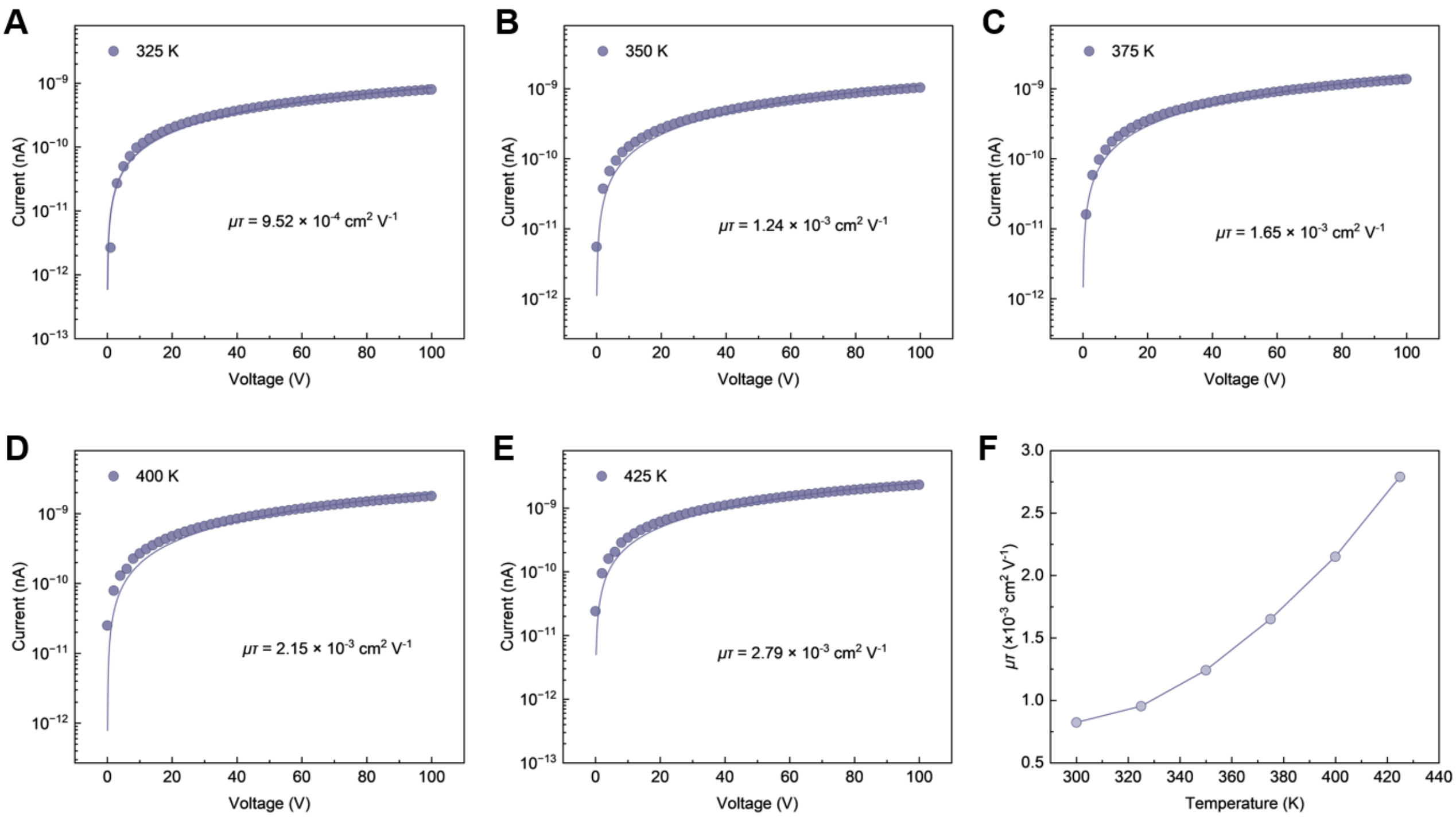

Fig. S17. The $\mu\tau$ value of 4Br2FBZ-CAB at different temperatures.

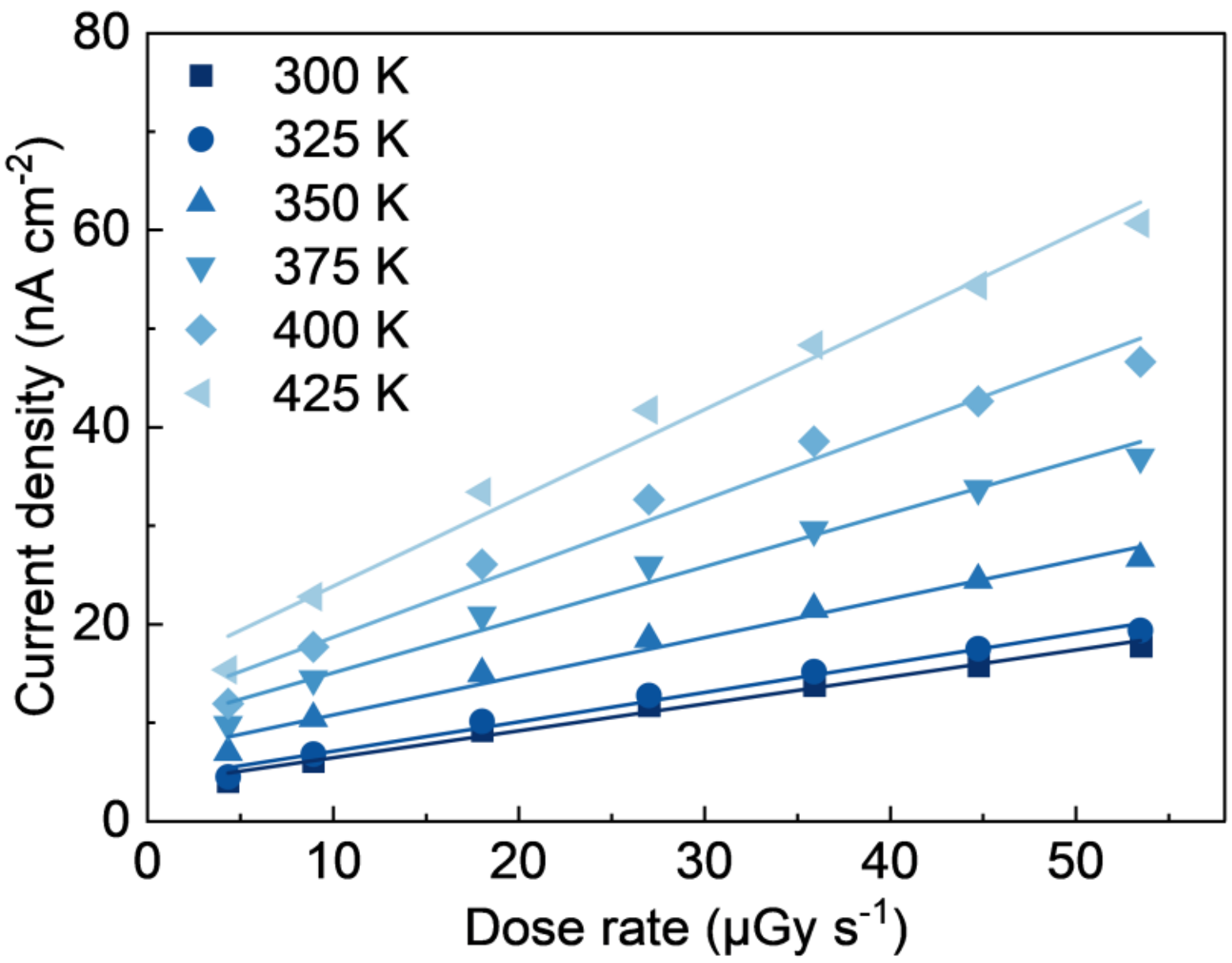

Fig. S18. X-ray generated current density versus dose rate under different temperatures.

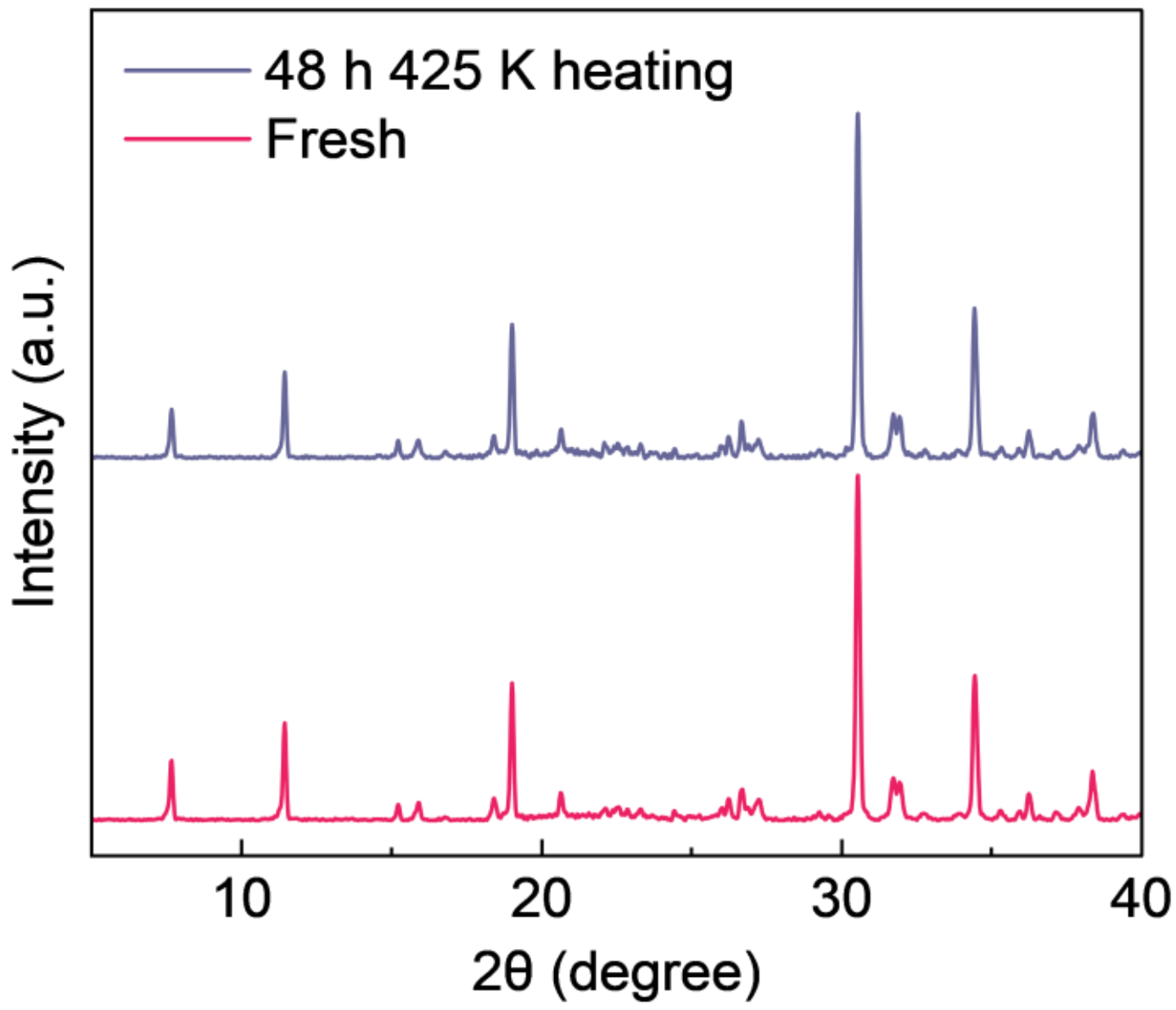

Fig. S19. PXRD patterns of 4Br2FBZ-CAB before and after thermal treatment at 425 K for 48 h.

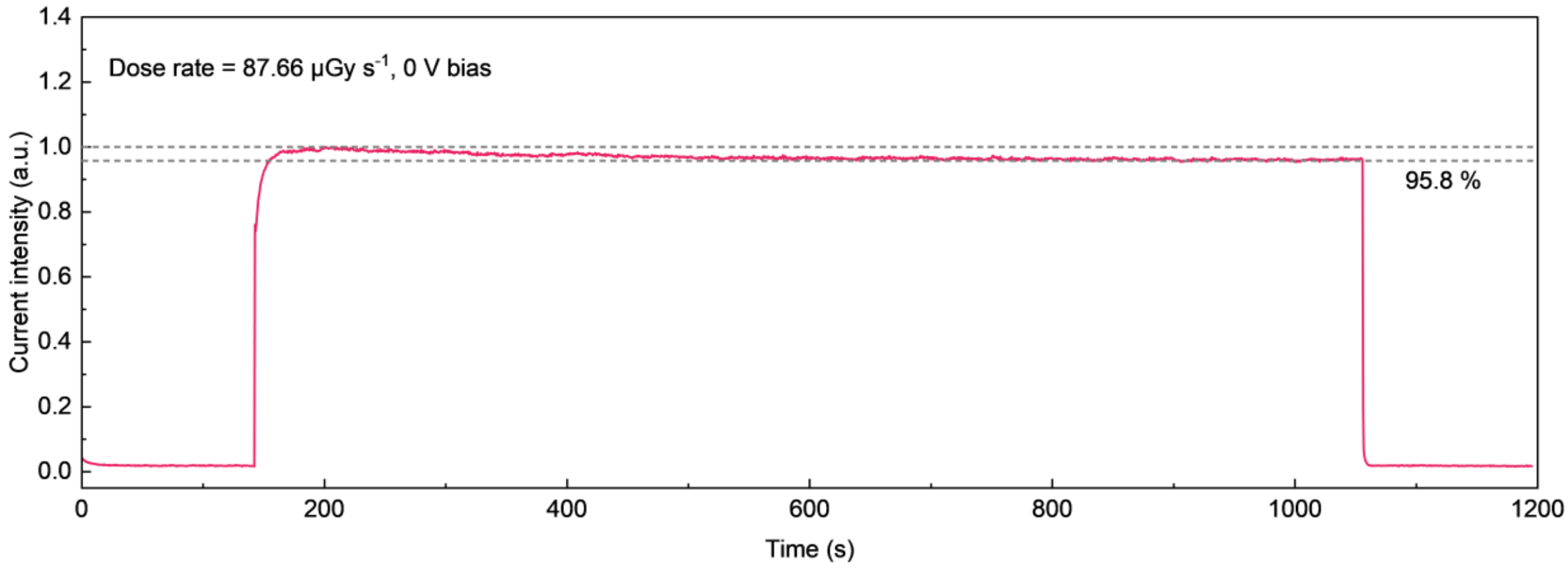

Fig. S20. Irradiation stability of the device at 425 K under 0 V bias.

Table S1. Crystal data and structure refinement.

**Compounds**	**PA-CAB**	**BA-CAB**	**BZ-CAB**	**4BrBZ-CAB**	**4Br2FBZ-CAB**
CCDC code	2101483	1814799	1998799	2215162	2422689
Empirical formula	$C_6H_{20}AgBiBr_7CsN_2$	$C_8H_{24}AgBiBr_7CsN_2$	$C_{14}H_{20}AgBiBr_7CsN_2$	$C_{14}H_{18}AgBiBr_9CsN_2$	$C_{14}H_{14}AgBiBr_9CsF_2N_2$
Formula weight	1129.37	1157.42	1225.45	1383.25	1417.22
Temperature/K	200.15	199.98	298.02	150.01	273.15
Crystal system	monoclinic	monoclinic	monoclinic	orthorhombic	orthorhombic
Space group	$P2_1$	$P2_1$	$P2_1$	$Abm2$	$Cmc2_1$
a/Å	8.0477(9)	8.0618(5)	8.0393(5)	7.9755(4)	47.057(6)
b/Å	7.8998(9)	7.8928(4)	7.9337(4)	44.784(3)	8.0870(7)
c/Å	18.591(2)	19.7697(12)	22.2165(13)	7.9896(4)	7.9206(7)
α/°	90	90	90	90	90
β/°	102.410(4)	101.734(2)	100.340(2)	90	90
γ/°	90	90	90	90	90
Volume/Å^3	1154.3(2)	1231.66(12)	1393.99(14)	2853.7(3)	3014.2(5)
Z	2	2	2	4	4
ρ_{calc} (g/cm^3)	3.249	3.121	2.920	3.220	3.123
μ/mm^{-1}	22.118	20.734	18.329	20.711	19.621
F(000)	1000.0	1032.0	1096.0	2464.0	2520.0
Radiation	MoKα (λ = 0.71073)	MoKα (λ = 0.71073)	MoKα (λ = 0.71073)	MoKα (λ = 0.71073)	MoKα (λ = 0.71073)
2Θ range for data collection/°	5.156 to 49.994	5.162 to 49.974	5.134 to 54.992	5.108 to 49.986	5.11 to 50
Index ranges	$-9 \le h \le 9$, $-9 \le k \le 9$, $-22 \le l \le 22$	$-9 \le h \le 9$, $-9 \le k \le 9$, $-23 \le l \le 23$	$-10 \le h \le 10$, $-10 \le k \le 10$, $-28 \le l \le 28$	$-9 \le h \le 9$, $-50 \le k \le 52$, $-9 \le l \le 9$	$-55 \le h \le 55$, $-9 \le k \le 9$, $-9 \le l \le 9$
Reflections collected	10774	18268	21917	8055	8200
Independent reflections	3980 [R_{int} = 0.0629, R_{sigma} = 0.0773]	4363 [R_{int} = 0.0517, R_{sigma} = 0.0416]	6386 [R_{int} = 0.0774, R_{sigma} = 0.0837]	2535 [R_{int} = 0.0443, R_{sigma} = 0.0550]	2367 [R_{int} = 0.0854, R_{sigma} = 0.0933]
Data/restraints/parameters	3980/94/168	4363/52/186	6386/97/215	2535/141/131	2367/63/129
Goodness-of-fit on F^2	1.119	1.061	1.092	1.268	1.094
Final R indexes [I>=2σ (I)]	R_1 = 0.0646, wR_2 = 0.1830	R_1 = 0.0358, wR_2 = 0.0748	R_1 = 0.0505, wR_2 = 0.1155	R_1 = 0.0793, wR_2 = 0.1948	R_1 = 0.0922, wR_2 = 0.2222
Final R indexes [all data]	R_1 = 0.0746, wR_2 = 0.1922	R_1 = 0.0394, wR_2 = 0.0764	R_1 = 0.0627, wR_2 = 0.1217	R_1 = 0.0814, wR_2 = 0.1961	R_1 = 0.1078, wR_2 = 0.2312
Largest diff. peak/hole / e Å^{-3}	4.33/-3.45	1.86/-1.29	3.19/-2.65	3.69/-4.75	3.29/-2.52

Table S2. Bond Lengths for 4Br2FBZ-CAB.

Atom	Atom	Length/Å	Atom	Atom	Length/Å
(Ag/Bi)1	Br3[1]	2.810(8)	C2	C7	1.3900
(Ag/Bi)1	Br3	2.915(8)	C2	C3	1.3900
(Ag/Bi)1	Br2	2.824(8)	C2	C1	1.391(8)
(Ag/Bi)1	Br2[2]	2.828(8)	C7	C6	1.3900
(Ag/Bi)1	Br4	2.830(3)	C7	F1	1.30(4)
(Ag/Bi)1	Br1	2.850(6)	C6	C5	1.3900
Br5	C5	1.89(3)	C5	C4	1.3900
N1	C1	1.401(13)	C4	C3	1.3900

Table S3. Bond Angles for 4Br2FBZ-CAB.

Atom	Atom	Atom	Angle/°	Atom	Atom	Atom	Angle/°
Br3^{1}	(Ag/Bi)1	Br3	87.53(6)	(Ag/Bi)1^{5}	Br3	(Ag/Bi)1	174.1(2)
Br3^{1}	(Ag/Bi)1	Br2	175.33(17)	(Ag/Bi)1	Br2	(Ag/Bi)1^{7}	164.8(2)
Br3^{1}	(Ag/Bi)1	Br2^{2}	91.3(4)	(Ag/Bi)1	Br4	(Ag/Bi)1^{6}	166.7(3)
Br3^{1}	(Ag/Bi)1	Br4	87.3(3)	C2	C1	N1	118.4(15)
Br3^{1}	(Ag/Bi)1	Br1	90.97(18)	C7	C2	C3	120.0
Br2	(Ag/Bi)1	Br3	91.9(4)	C7	C2	C1	119.7(8)
Br2^{2}	(Ag/Bi)1	Br3	175.1(2)	C3	C2	C1	120.3(8)
Br2	(Ag/Bi)1	Br2^{2}	88.96(6)	C6	C7	C2	120.0
Br2	(Ag/Bi)1	Br4	88.1(3)	F1	C7	C2	115(3)
Br2^{2}	(Ag/Bi)1	Br4	86.0(3)	F1	C7	C2	125(3)
Br2	(Ag/Bi)1	Br1	93.71(19)	C7	C6	C5	120.0
Br2^{2}	(Ag/Bi)1	Br1	88.77(19)	C6	C5	Br5	115(2)
Br4	(Ag/Bi)1	Br3	89.2(3)	C4	C5	Br5	125(2)
Br4	(Ag/Bi)1	Br1	174.4(4)	C4	C5	C6	120.0
Br1	(Ag/Bi)1	Br3	96.01(18)	C3	C4	C5	120.0
				C4	C3	C2	120.0

Table S4. Comparison of refinement results using different occupancy models for the metal site.

Model	R_1	wR_2	Largest diff. peak/ hole /e Å^{-3}	Occupancy (Ag/Bi)
Constrained Ag:Bi = 0.5:0.5	0.0922	0.2222	3.29/-2.52	0.5/0.5
Free refinement of Ag/Bi	0.0922	0.2305	3.2/-2.6	0.49(2)/0.51(2)
Ag-only (Bi = 0)	0.1221	0.3385	5.9/-2.5	1/0
Bi-only (Ag = 0)	0.1083	0.3111	3.4/-5.4	0/1

Table S5. Structural information of the compounds and calculated distortions of the metal halide octahedron.

Compounds	Space group	Crystal system	Solid solution of Ag and Bi ions	Halide octahedra	Δd ($\times 10^{-4}$)	σ_1^2	σ_2^2
$(4Br2FBZ)_2CsAgBiBr_7$	*Cmc*2$_1$	orthorhombic	yes	[Ag/BiBr$_6$]	1.461	8.3683	0.2171
$(4BrBZ)_2CsAgBiBr_7$	*Abm*2	orthorhombic	yes	[Ag/BiBr$_6$]	0.9150	6.1611	0.9737
$(BZ)_2CsAgBiBr_7$	*P*2$_1$@LTP	monoclinic	no	[AgBr$_6$]	15.8	48.9679	16.6430
				[BiBr$_6$]	1.268	4.1757	5.5953
	*I*4/*mmm*@HTP	tetragonal	yes	[Ag/BiBr$_6$]	0.378	1.2341	2.2585
$(BA)_2CsAgBiBr_7$	*P*2$_1$@LTP	monoclinic	no	[AgBr$_6$]	27.1	89.0744	45.7418
				[BiBr$_6$]	4.184	6.1161	4.7694
	*P*2$_1$/*m*@ITP	monoclinic	no	[AgBr$_6$]	6.202	23.5410	22.3150
				[BiBr$_6$]	1.241	2.1858	0.0097
	*I*4/*mmm*@HTP	tetragonal	yes	[Ag/BiBr$_6$]	0.203	3.0577	5.5597
$(PA)_2CsAgBiBr_7$	*P*2$_1$@LTP	monoclinic	no	[AgBr$_6$]	18.3	56.5765	53.2911
				[BiBr$_6$]	0.771	1.3886	1.2337
	*P*2$_1$/*m*@HTP	monoclinic	no	[AgBr$_6$]	13.5	39.11011	27.3733
				[BiBr$_6$]	0.309	0.9896	0.4832
$(4Br2FBZ)_4AgBiBr_8$	*Cmc*2$_1$	orthorhombic	yes	[Ag/BiBr$_6$]	8.303	1.0042	0.3081
$(BZ)_4AgBiBr_8$	*P*2$_1$/*c*@LTP	monoclinic	no	[AgBr$_6$]	20.6	2.3698	0
				[BiBr$_6$]	1	3.6613	0
	Cmce@HTP	orthorhombic	yes	[Ag/BiBr$_6$]	5.251	0.0704	0
$(BA)_4AgBiBr_8$	*P*2$_1$/*c*@LTP	monoclinic	no	[AgBr$_6$]	26.5	3.4578	0
				[BiBr$_6$]	0.565	6.0580	0
	*C*2/*m*@HTP	monoclinic	no	[AgBr$_6$]	51.6	8.2601	0
				[BiBr$_6$]	0.035	0.5119	0
$(PA)_4AgBiBr_8$	*C*2/*m*	monoclinic	no	[AgBr$_6$]	31.6	13.0112	0
				[BiBr$_6$]	0.263	0.6393	0

Table S6. Performance comparison of the 4Br2FBZ-CAB X-ray detector and some reported X-ray detectors.

Dimensionality	Materials	Sensitivity ($\mu C\ Gy^{-1}\ cm^{-2}$)	Detection limit ($nGy\ s^{-1}$)	Condition	Ref.
2D	4Br2FBZ-CAB	896.9	13.9	0 V	This work
		3098.2	478	90 V	
	$(BA)_2(EA)_2Pb_3I_{10}$	391.6	17.2	0 V	(*73*)
	(R-MPA)(BrEA)$PbBr_4$	84.9	21.7	0 V	(*56*)
	$(NPA)_2(EA)_2Pb_3Br_{10}$	225	83.4	0 V	(*74*)
	$(BrPA)_2(EA)_2Pb_3Br_{10}$	562.6	118	0 V	(*54*)
	(R-MPA)$_4AgBiI_8$	46.3	85	0 V	(*76*)
	$(DFPIP)_4AgBiI_8$	188	3130	50 V	(*16*)
	$(BDA)PbI_4$	242	430	494 V/mm	(*70*)
	$(BA)_2PbI_4$	148	241	10 V/mm	(*69*)
	$(PMA)_2PbI_4$	283	2130	50 V	(*71*)
	$(HA)PbI_4$	293	51.38	0 V	(*75*)
	$(F\text{-}PEA)_2PbI_4$	3402	23	200 V	(*67*)
	$(2IPA)_2FAPb_2I_7$	438	20	360 V/mm	(*68*)
	$(NH_4)_3Bi_2I_9$	8200	55	10 V/mm	(*66*)
1D/0D	$(HDA)BiI_5$	170.7	266	0 V	(*79*)
	$(BZA)_2$(R/S-PPA)BiI_6	268.2	133	5 V	(*72*)
	(R/S-PPA)$_2BiI_5$	31	270	0 V	(*77*)
	(1,3-BMACH)BiI_5	170	49	0 V	(*78*)
3D	$Cs_2AgBiBr_6$	1974	45.7	50 V/mm	(*65*)
	$GAMAPbI_3$	23100	16.9	5 V	(*64*)
	$CsPbBr_3$	41180	10.81	1000 V	(*63*)
	$MhyPbBr_3$	220	203	0 V	(*59*)
		514	5428	100 V	
heterojunction	$Cs_2AgBiBr_6$/ $(BA)_2CsAgBiBr_7$	206	40000	0 V	(*80*)
	$MAPbI_3/(PEA)_2PbI_4$	291.8	500	0 V	(*60*)

Dimensionality	Materials	Sensitivity ($\mu C\ Gy^{-1}\ cm^{-2}$)	Detection limit ($nGy\ s^{-1}$)	Condition	Ref.
traditional	α-Se	20	5500	100000 V/cm	(*82*)
	HgI_2	1600	10000	2500 V/cm	(*81*)
	CdZnTe	2400	50000	2500 V/cm	(*83*)

Data S1. Crystal structure data. Crystal structure data for 4Br2FBZ-CAB measured at 273.15 K.
